# High selenium diet attenuates pressure overload-induced cardiopulmonary oxidative stress, inflammation, and heart failure

**DOI:** 10.1016/j.redox.2024.103325

**Published:** 2024-08-22

**Authors:** Umesh Bhattarai, Rui Xu, Xiaochen He, Lihong Pan, Ziru Niu, Dongzhi Wang, Heng Zeng, Jian-Xiong Chen, John S. Clemmer, Yingjie Chen

**Affiliations:** a Department of Physiology and Biophysics, School of Medicine, University of Mississippi Medical Center, Jackson, MS, United States; b Department of Pharmacology and Toxicology, School of Medicine, University of Mississippi Medical Center, Jackson, MS, United States

**Keywords:** Selenium, Pressure overload, Oxidative stress, Inflammation, Heart failure, T cells

## Abstract

Selenium (Se) deficiency is associated with the development of Keshan disease, a cardiomyopathy associated with massive cardiac immune cell infiltration that can lead to heart failure (HF). The purpose of this study was to determine whether high Se diet can attenuate systolic overload-induced cardiopulmonary inflammation and HF. Briefly, transverse aortic constriction (TAC)-induced cardiopulmonary oxidative stress, inflammation, left ventricular (LV) dysfunction, and pulmonary remodeling were determined in male mice fed with either high Se diet or normal Se diet. High Se diet had no detectable effect on LV structure and function in mice under control conditions, but high Se diet significantly protected mice from TAC-induced LV hypertrophy, dysfunction, increase of lung weight, and right ventricular hypertrophy. As compared with mice treated with normal Se diet, high Se diet also reduced TAC-induced LV cardiomyocyte hypertrophy, fibrosis, leukocyte infiltration, pulmonary inflammation, pulmonary fibrosis, and pulmonary micro-vessel muscularization. In addition, high Se diet significantly ameliorated TAC-induced accumulation and activation of pulmonary F4/80^+^ macrophages, and activation of dendritic cells. Interestingly, high Se diet also significantly attenuated TAC-induced activation of pulmonary CD4^+^ and CD8^+^ T cells. Moreover, we found that TAC caused a significant increase in cardiac and pulmonary ROS production, increases of 4-hydroxynonenal (4-HNE) and 3-nitrotyrosine (3-NT), as well as a compensatory increases of LV glutathione peroxidase 1 (GPX1) and 4 (GPX4) in mice fed with normal Se diet. Above changes were diminished in mice fed with high Se diet. Collectively, these data demonstrated that high Se diet significantly attenuated systolic pressure overload-induced cardiac oxidative stress, inflammation, HF development, and consequent pulmonary inflammation and remodeling.

## Highlights

• TAC increases cardiopulmonary oxidative stress and cardiac selenoproteins GPX1 and GPX4.
• Selenium supplement attenuates TAC-induced cardiopulmonary ROS and inflammation.
• Selenium supplement attenuates TAC-induced pulmonary APC and T cell activation.
• Selenium supplement attenuates TAC-induced HF development and progression.

## Graphical abstract

**Figure:**
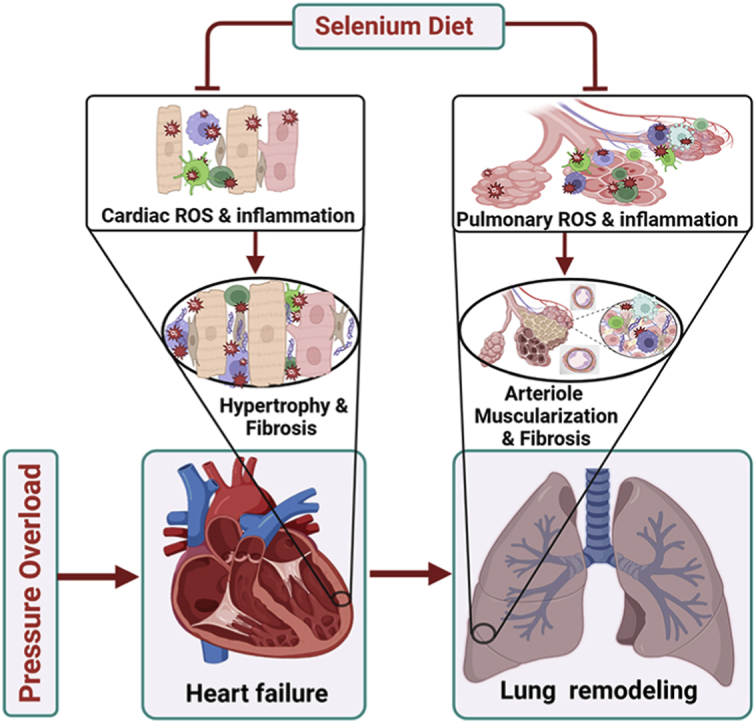

## 1 Introduction

Heart failure (HF) is a pathological condition in which the heart is unable to pump enough oxygen-rich blood to meet the body's needs. HF is one of the major causes of hospitalization, morbidity, and mortality worldwide [1]. After the development of HF, the pathological condition can often progress to pulmonary inflammation and remodeling, right ventricular (RV) hypertrophy, and RV failure [[2], [3], [4], [5], [6]]. This transitional process from LV failure to pulmonary remodeling, RV hypertrophy, and RV failure is often termed HF progression [6,7]. Leading causes of HF include coronary disease, high blood pressure, cardiac valve diseases, myocarditis, and congenital heart diseases. While the underlying mechanism of HF development varies, cardiac oxidative stress and inflammation have been recognized as important culprits for HF development and HF progression [8]. The bioavailability of minerals such as zinc, magnesium, and Selenium (Se) play a significant role in cardiopulmonary oxidative stress and inflammatory responses [9].

While inflammation is an important protective pathophysiological response against tissue damage and microbial invasion, dysregulated inflammatory responses can promote HF development and HF progression [6,10]. Specifically, cardiac inflammation can directly promote cardiomyocyte injury and dysfunction, while pulmonary inflammation exacerbates HF-induced pulmonary injury, fibrosis, micro-vessel remodeling, and the consequent RV hypertrophy and failure [3,6,11,12]. For example, studies have illustrated the important roles of interferon-gamma (IFN-γ), tumor necrosis factor-α (TNF-α), interleukin-1 beta (IL-1β), and interleukin-12 (IL-12) in cardiac inflammation and HF development, as well as HF-induced lung inflammation and remodeling [[12], [13], [14]]. Commonly recognized immune cell subsets include neutrophils, macrophages, B cells, T helper cells, cytotoxic T cells, dendritic cells, and natural killer (NK) cells, which can be identified by their unique cellular markers. For example, all immune cells are CD45^+^ cells, B cells express CD19 and CD20, cytotoxic T cells express CD8, helper T cells express CD4, NK cells express NK1.1 and NKp46. These immune cellular markers are often used to determine the immune cell activation and functional capacities by flow cytometry. Previous studies from us and others have demonstrated that immune cell subsets, such as regulatory T cells (Tregs, Tregs generally express Foxp3 and CD25), CD4^+^ T cells, CD11c^+^ antigen-presenting cells play important roles in HF development and progression [3,7,[15], [16], [17]]. We also found that inhibition of T cell activation effectively protects the heart against TAC-induced cardiac inflammation and HF development and/or HF progression [3,7]. Previous studies also demonstrate an important role of oxidative stress in promoting HF development [[18], [19], [20], [21], [22]] and HF-induced pulmonary inflammation and remodeling [11,12,23]. Consistent with experimental studies, clinical studies have similarly demonstrated that inflammation is closely interrelated to HF development and HF outcomes [10,24].

Se is an important micronutrient with multiple biological functions. One of the major biological effects of Se is through the formation of selenocysteine by replacing the Sulphur in cysteine. Selenocysteine is further incorporated within Se-containing proteins (selenoproteins) to protect redox-sensitive biological pathways [25,26]. Studies have demonstrated that Se deficiency in humans is associated with a deadly form of dilated cardiomyopathy called Keshan disease, a disease associated with massive cardiac immune cell infiltration and high incidence of HF [27]. Se deficiency also exacerbates chronic myocarditis in coxsackievirus-infected mice likely through promoting viral genome mutations and virulence of coxsackievirus [[28], [29], [30]]. In addition, several retrospective clinical studies demonstrated that Se deficiency in HF patients is associated with impaired exercise tolerance and a higher mortality rate [9,31,32], while Se supplementation is effective in treating cardiomyopathy in patients [33,34]. Moreover, Se and selenoproteins regulate proliferation and activation of CD4^+^ T cells, CD8^+^ T cells, and NK cells [35], immune cell subsets that have been shown to contribute to HF development [16,17]. While Se deficiency is associated with cardiac immune cell dysfunction and HF [27], the role of high Se diet on systolic overload-induced cardiac inflammation, hypertrophy, and HF development during conditions without Se deficiency is unknown.

To test the hypothesis that increased oxidative stress during HF development requires more Se to overcome cardiopulmonary oxidative stress, we studied the effect of high Se diet and normal Se diet on transverse aortic constriction (TAC)-induced HF development and HF progression in mice. Interestingly, as compared with mice treated with normal Se diet, TAC-induced cardiopulmonary oxidative stress and inflammation, LV dysfunction, pulmonary fibrosis, and pulmonary micro-vessel remodeling were significantly reduced in mice fed with high Se diet. The results represent an important step toward understanding the effects of a high Se diet on systolic overload-induced cardiopulmonary oxidative stress and inflammation during HF development.

## 2 Materials and methods

**Detailed methods** are available in the online-only Data Supplement.

**Animals and Experimental protocols:** Male C57BL/6J mice were purchased from Jackson Laboratory (Bar Harbor, ME) and were housed in ventilated cages in a temperature-controlled environment with 12-h light/dark cycles. The mice were randomly divided into two groups and fed with standard chow diet containing normal Se (80 ppb Se, Catalog # TD.07325) or high Se (400 ppb Se, Catalog # TD.07326) (Envigo RMS Holding Corp.) according to the protocol used by Dr. K. Sandeep Prabhu's group [36,37]. One week after either normal or high Se diets, mice were subjected to either sham surgery or TAC [38,39], a commonly used surgical procedure to generate systolic overload to mimic clinical conditions such as aortic stenosis or hypertension. The TAC surgery was performed in mice after anesthesia with intraperitoneal injection of Ketamine (100 mg/kg) and xylazine (10 mg/kg) [7,17,38,39]. A final cardiac functional test was performed eight weeks after the surgery, then followed with sample collection and subsequent histological and biochemical analyses as detailed in the online-only Data Supplement. Since TAC-induced cardiac immune cell infiltration peaks at ∼7 days after TAC, to determine the effect of high Se diet on early-phase of TAC-induced cardiac inflammation, oxidative stress, dysfunction, additional mice fed with normal or high Se diets were also subjected to TAC, then followed with sample collection at 7 days after TAC. The experimental studies were approved by the Institutional Animal Care and Use Committee at the University of Mississippi Medical Center.

**Statistical Analyses:** Data were presented as mean ± SEM. A two-way ANOVA followed by the Bonferroni post-hoc test was used to test the statistical differences among the sham and TAC groups fed with normal or high Se diet using GraphPad Prism 9 software. Unpaired *t*-test was used to determine statistical differences between two groups. Survival data were shown as Kaplan-Meier curves. Log-rank test was used to analyze survival curves. p < 0.05 was considered statistically significant.

## 3 Results

**Fig. 1:**
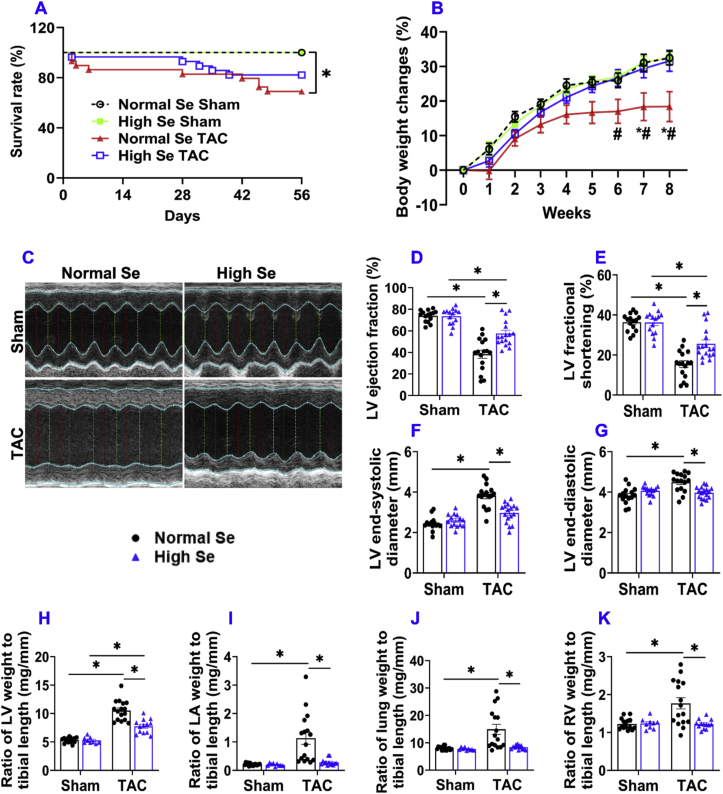
**High Se diet attenuated chronic TAC-induced cardiac dysfunction and increase in LV weight, LA weight, lung weight, and RV weight.** (A) Survival curves of mice fed with normal or high Se TAC (log-rank test). (B) Percentage of body weight changes during the post-TAC study period. (C) Representative M-mode echocardiography images of the indicated groups. (D–G) Quantified data of echocardiographic measurements of LV ejection fraction, LV fractional shortening, LV end-systolic diameter, and LV end-diastolic diameter. (H–K) The ratio of LV weight, LA weight, lung weight, and RV weight to tibial length. *p < 0.05; *Statistics for Normal Se Sham vs Normal Se TAC; #Statistics for Normal Se TAC vs High Se TAC; Se, Selenium; n = 9 to17 per group.

**Fig. 2:**
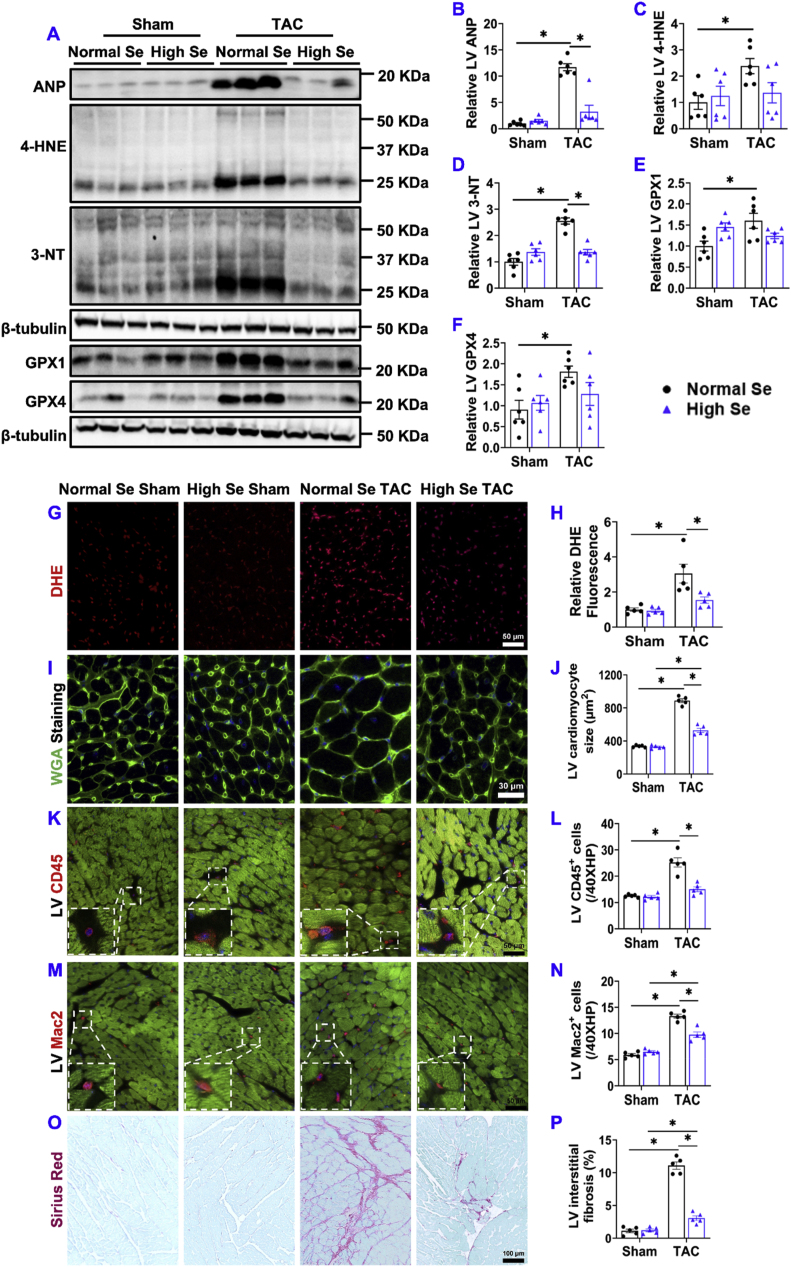
**High Se diet significantly attenuated chronic TAC-induced LV oxidative stress, cardiomyocyte hypertrophy, inflammation, and fibrosis.** (A–F) Representative western blots and quantification of LV atrial natriuretic peptide (ANP), 4-hydroxynonenal (4-HNE), 3-nitrotyrosine (3-NT), glutathione peroxidase 1 (GPX1), and glutathione peroxidase 4 (GPX4) of the indicated groups. (G, H) Representative dihydroethidium (DHE) staining images and quantified data of DHE intensity of the indicated groups. (I, J) Representative wheat germ agglutinin (WGA) staining images and quantified data of LV cardiomyocyte cross-sectional area of the indicated groups. (K–N) Representative images and quantified data of LV CD45^+^ leukocytes and Mac2^+^ leukocytes infiltration performed by immuno-histological staining. (O, P) Representative images and quantified data of LV interstitial fibrosis performed by Sirius red/Fast green staining. *p < 0.05; Se, Selenium; n = 5 to 6 per group. (For interpretation of the references to colour in this figure legend, the reader is referred to the Web version of this article.)

**Fig. 3:**
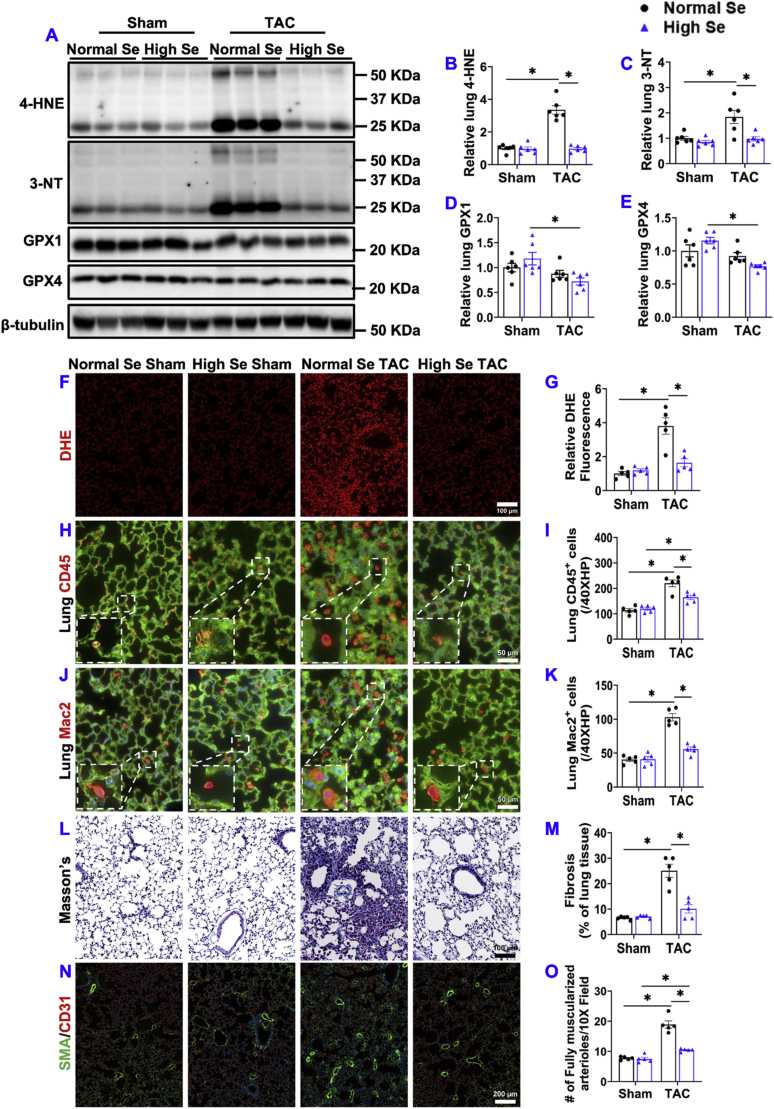
**High Se diet significantly attenuated chronic TAC-induced pulmonary oxidative stress, inflammation, fibrosis, and vessel remodeling.** (A–E) Representative western blots and quantification of lung 4-hydroxynonenal (4-HNE), 3-nitrotyrosine (3-NT), glutathione peroxidase 1 (GPX1), and glutathione peroxidase 4 (GPX4) of the indicated groups. (F, G) Representative images of dihydroethidium (DHE) staining and quantified data of DHE staining of the indicated groups. (H–K) Representative images and quantified data of pulmonary infiltration of CD45^+^ leukocytes and Mac2^+^ leukocytes performed by immuno-histological staining. (L, M) Representative images and quantified data of pulmonary fibrosis performed by Masson's trichrome staining. (N, O) Representative images and quantified data pulmonary vessel remodeling performed by immuno-histological staining. *p < 0.05; Se, Selenium; n = 5 to 6 per group.

**Fig. 4:**
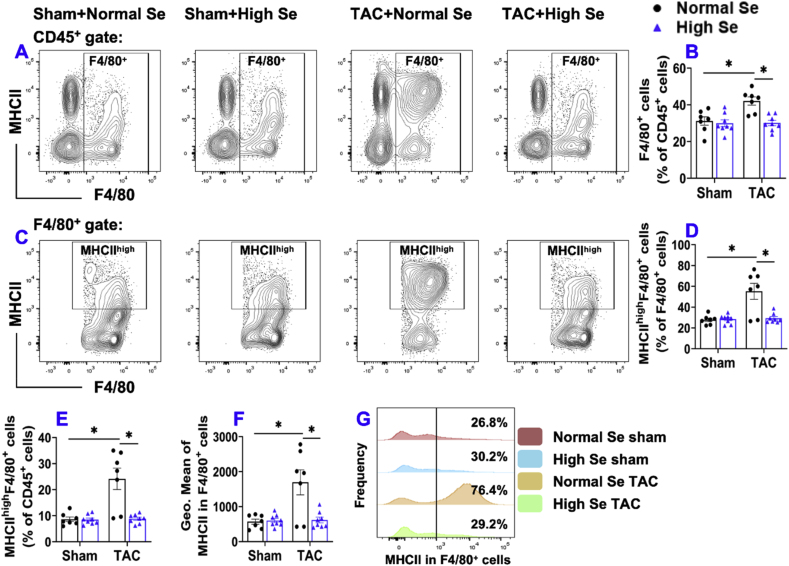
**High Se diet significantly attenuated chronic TAC-induced pulmonary F4/80**^**+**^**macrophage accumulation and activation.** (A) Flow cytometry plots of lung F4/80^+^ macrophages. (B) Quantified data of the percentage of F4/80^+^ cells within CD45^+^ cells. (C) Flow cytometry plots used for the detection of MHCII expression in F4/80^+^ cells. (D, E) Quantified data of the percentages of MHCII^high^F4/80^+^ cells within F4/80^+^ and CD45^+^ cells. (F) Quantified data of mean fluorescent intensity of MHCII in F4/80^+^ cells. (G) Representative histograms of MHCII expression in F4/80^+^ cells of the indicated groups. *p < 0.05; Se, Selenium; n = 7 to 8 per group.

**Fig. 5:**
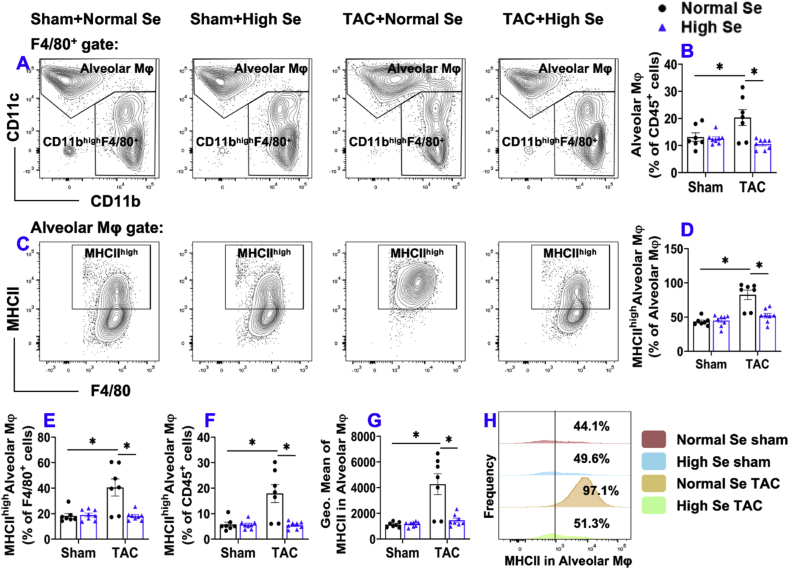
**High Se diet significantly attenuated chronic TAC-induced accumulation and activation of pulmonary alveolar macrophages (CD11c**^**high**^**CD11b**^**low**^**F4/80**^**+**^**).** (A) Flow cytometry plots of lung alveolar macrophages. (B) Quantified data of the percentage of alveolar macrophages within CD45^+^ cells. (C) Flow cytometry plots used for the detection of MHCII expression in alveolar macrophages. (D–F) Quantified data of the percentages of MHCII^high^ alveolar macrophages within alveolar macrophages, F4/80^+^, and CD45^+^ cells, respectively. (G) Quantified data of mean fluorescent intensity of MHCII in alveolar macrophages. (H) Representative histograms of MHCII expression in alveolar macrophages of the indicated groups. *p < 0.05; Se, Selenium; Mφ, macrophages; n = 7 to 8 per group.

**Fig. 6:**
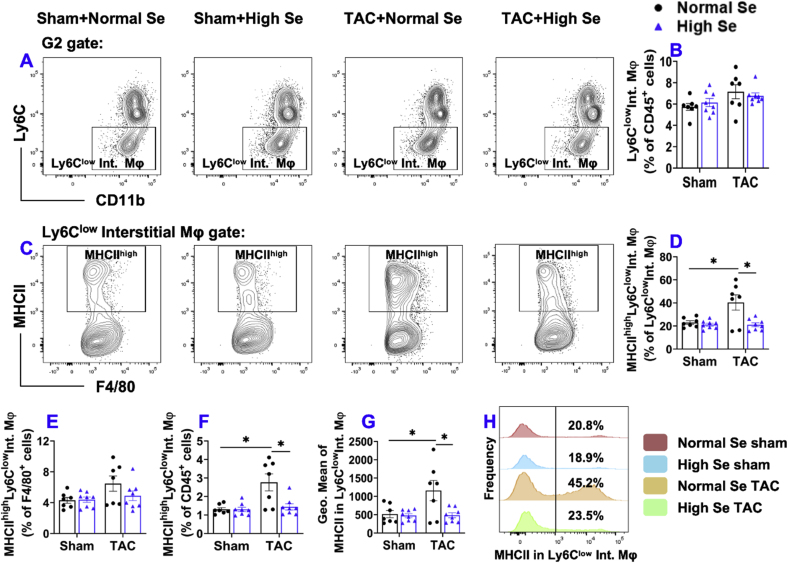
**High Se diet significantly attenuated chronic TAC-induced activation of pulmonary Ly6C**^**low**^**interstitial macrophages (Ly6C**^**low**^**CD11c**^**low**^**CD11b**^**high**^**F4/80**^**+**^**).** (A) Flow cytometry plots of lung Ly6C^low^ interstitial macrophages. (B) Quantified data of the percentage of Ly6C^low^ interstitial macrophages within CD45^+^ cells. (C) Flow cytometry plots used for the detection of MHCII expression in Ly6C^low^ interstitial macrophages. (D–F) Quantified data of the percentages of MHCII^high^Ly6C^low^ interstitial macrophages within Ly6C^low^ interstitial macrophages, F4/80^+^, and CD45^+^ cells, respectively. (G) Quantified data of mean fluorescent intensity of MHCII in Ly6C^low^ interstitial macrophages. (H) Representative histograms of MHCII expression in Ly6C^low^ interstitial macrophages of the indicated groups. *p < 0.05; Se, Selenium; Mφ, macrophages; Int., interstitial; n = 7 to 8 per group.

1. **Chronic TAC-induced cardiac dysfunction was significantly attenuated in mice fed with high Se diet.** TAC significantly increased mortality rate in mice with normal Se diet but not in mice with high Se diet (Fig. 1A). However, the mortality rate between two TAC groups were not statistically different. The slightly but insignificant higher mortality in normal Se diet treated mice after TAC is likely due to their poor capacity to tolerate TAC procedure. Bodyweight gain after surgical procedure was significantly decreased in the mice fed with normal Se diet after TAC, but not in mice fed with high Se diet after TAC (Fig. 1B and Supplementary Table 2), indicating a better stress handling capacity in mice fed with high Se diet. Echocardiography showed that baseline LV ejection fraction and fractional shortening were similar in mice under control conditions after normal Se or high Se diet (Fig. 1C–E). TAC caused significant reduction of LV ejection fraction and LV fractional shortening in mice with both normal or high Se diet, but the TAC-induced decrease in LV ejection fraction and LV fractional shortening were significantly attenuated in mice after the high Se diet as compared with mice after normal Se diet (Fig. 1C–E). High Se diet also significantly attenuated TAC-induced increases of LV end-systolic diameter, LV end-diastolic diameter (Fig. 1C, F, G), LV end-systolic volume, and LV end-diastolic volume (Supplementary Fig. 2).

2. **High Se diet significantly attenuated chronic TAC-induced LV hypertrophy, increase of lung weight, and RV hypertrophy.** High Se diet had no detectable effect on LV weight, LA weight, lung weight, RV weight, and their ratios to body weight or tibial length under sham conditions in mice (Fig. 1H–K and Supplementary Table 2). However, high Se diet significantly attenuated 8 week's TAC-induced increases of LV weight, LA weight, lung weight, RV weight, and their ratios to bodyweight or tibial length in mice (Fig. 1H–K and Supplementary Table 2).
3. **High Se diet significantly attenuated chronic TAC-induced LV oxidative stress, cardiomyocyte hypertrophy, inflammation, and fibrosis.** Se exerts an important biological role in attenuating oxidative stress [40,41]. Since increased oxidative stress contributes to HF development [20,22,42]. We further determined the oxidative stress and the expression of selenoproteins glutathione peroxidase 1 (GPX1) and glutathione peroxidase 4 (GPX4). High Se diet significantly attenuated TAC-induced LV atrial natriuretic peptide (ANP), a biomarker for cardiac congestion and HF severity (Fig. 2A and B). In addition, high Se diet significantly attenuated TAC-induced expressions of LV 4-hydroxynonenal (4-HNE) and 3-nitrotyrosine (3-NT), two commonly used markers of oxidative stress (Fig. 2A, C, D). Interestingly, TAC caused significant increases of LV selenoproteins GPX1 and GPX4 in mice treated with normal Se diet but not in mice treated with high Se diet (Fig. 2A, E, F). In addition, dihydroethidium (DHE) staining showed that TAC-induced cardiac oxidative stress was also significantly attenuated in mice fed with high Se diet (Fig. 2G and H). Moreover, high Se diet significantly attenuated TAC-induced LV cardiomyocyte hypertrophy, LV accumulation of CD45^+^ leukocytes and Mac2^+^ leukocyte subset (Mac2 is generally expressed in some leukocytes such as macrophages), and LV interstitial and perivascular fibrosis (Fig. 2I–P and Supplementary Fig. 3).

4. **High Se diet significantly attenuated chronic TAC-induced pulmonary oxidative stress, inflammation, fibrosis, and vessel remodeling.** Since our previous studies have shown that HF causes lung oxidative stress and inflammation, while these changes contribute to HF progression and RV hypertrophy [6,11,12], we further determined the effect of high Se diet on TAC-induced pulmonary oxidative stress, leukocyte infiltration, fibrosis, and vascular remodeling. High Se diet significantly attenuated TAC-induced expressions of pulmonary 4-HNE and 3-NT (Fig. 3A–C). Surprisingly, pulmonary GPX1 and GPX4 expressions were significantly decreased in mice with high Se diet after TAC as compared to corresponding sham mice (Fig. 3A, D, E). In addition, TAC also caused a significant increase in pulmonary oxidative stress in mice fed with normal Se, while TAC-induced pulmonary oxidative stress was abolished in mice fed with high Se diet (Fig. 3F and G). Pulmonary CD45^+^ and Mac2^+^ leukocytes were significantly increased after TAC in mice fed with normal Se, while the above changes were significantly attenuated in mice fed with high Se diet (Fig. 3H–K). In addition, high Se diet attenuated TAC-induced pulmonary fibrosis (Fig. 3L and M) and muscularization of pulmonary arterioles (Fig. 3N and O).

5. **High Se diet significantly attenuated chronic TAC-induced pulmonary F4/80**^**+**^**macrophage accumulation and activation.** Since we previously showed that pulmonary macrophages, the most populated immune cell subset in lung tissue, were significantly increased after HF development [3,6,14], we further determined pulmonary macrophage subsets by detecting F4/80^+^ immune cells in CD45^+^ leukocytes. The percentage of pulmonary F4/80^+^ macrophages in CD45^+^ leukocytes was significantly increased in normal Se-fed mice after TAC, while high Se diet significantly attenuated TAC-induced increase of the percentage of pulmonary F4/80^+^ macrophages (Fig. 4A and B). Since MHCII expression in macrophages reflects macrophage polarization and activation, MHCII expression in pulmonary macrophages was determined (Fig. 4C). The percentage of MHCII^high^F4/80^+^ macrophages within F4/80^+^ macrophages and CD45^+^ leukocytes were significantly increased after TAC in normal Se-fed mice, and these changes were abolished in mice after the high Se diet (Fig. 4D and E). In addition, the TAC-induced increase of average MHCII expression in F4/80^+^ macrophages (as indicated by GEO mean of MHCII) was also significantly reduced in mice after high Se diet (Fig. 4F). Moreover, high Se diet also significantly decreased the frequency of pulmonary MHCII^high^F4/80^+^ macrophages in mice (Fig. 4G). The percentages of neutrophils (Ly6G^++^CD11b^+^), B cells (CD19^+^MHCII^+^ cells), and NK1.1 cells (generally include NK and NK T cells) in total CD45^+^ leukocytes were not significantly different among these experimental groups (Supplementary Fig. 4).

6. **High Se diet significantly attenuated chronic TAC-induced alterations of pulmonary macrophage subsets and their activation.** Since pulmonary macrophages can be further grouped to macrophage subsets according to their expression of CD11c, CD11b, Ly6C, and MHCII. Specifically, pulmonary macrophages were further classed as alveolar macrophages according their high CD11c expression (CD11c^high^CD11b^low^F4/80^+^) (Fig. 5), Ly6C^low^ interstitial macrophages (Ly6C^low^CD11c^low^CD11b^high^F4/80^+^) (Fig. 6), and monocytes-derived Ly6C^high^ interstitial macrophages (Ly6C^high^CD11c^low^CD11b^high^F4/80^+^) (Supplementary Fig. 5) [43,44].

As presented in Fig. 5, the percentages of CD11c^high^ alveolar macrophages within CD45^+^ cells were significantly increased after TAC in mice fed with normal Se, while high Se diet abolished these changes (Fig. 5A and B). The percentages of MHCII^high^ alveolar macrophages within alveolar macrophages, F4/80^+^ macrophages, and CD45^+^ leukocytes were significantly increased after TAC in mice fed with normal Se, while high Se diet abolished these changes (Fig. 5C-F). In addition, high Se diet significantly abolished the TAC-induced increase of MHCII expression in alveolar macrophages (Fig. 5G). Histogram shows that the frequency of MHCII^high^ alveolar macrophages was significantly increased after TAC in the mice fed with normal Se, while high Se diet abolished these changes (Fig. 5H).

As shown in Fig. 6, the percentages of Ly6C^low^ interstitial macrophages within CD45^+^ leukocytes were not significantly different between sham and TAC group with or without high Se diet (Fig. 6A and B). We also quantified MHCII expression in Ly6C^low^ interstitial macrophages to determine the activation status of these macrophages (Fig. 6C). The percentages of MHCII^high^Ly6C^low^ interstitial macrophages within Ly6C^low^ interstitial macrophages and CD45^+^ leukocytes were significantly increased after TAC in normal Se fed mice, while high Se diet abolished the increase (Fig. 6D and F). Moreover, the average expression of MHCII in Ly6C^low^ interstitial macrophages was significantly increased after TAC in mice fed with normal Se diet, while high Se diet abolished the TAC-induced increase of MHCII expression in Ly6C^low^ interstitial macrophages (Fig. 6G). Histogram shows that the frequency of MHCII^high^Ly6C^low^ interstitial macrophages was significantly increased after TAC in mice fed with normal Se diet, while high Se diet abolished these changes (Fig. 6H).

Ly6C^high^CD11c^low^CD11b^high^F4/80^+^ macrophages are generally regarded as monocyte-derived interstitial macrophages. As presented in Supplementary Fig. 5, the percentages of Ly6C^high^ interstitial macrophages within CD45^+^ leukocytes were not different in sham or TAC group, with or without high Se diet (Supplementary Figs. 5A and B). The percentages of MHCII^high^Ly6C^high^ interstitial macrophages within Ly6C^high^ interstitial macrophages, F4/80^+^ macrophages, and CD45^+^ leukocytes were all significantly increased after TAC in normal Se fed mice (Supplementary Figs. 5C–F), while high Se diet completely abolished TAC-induced increases in the percentage of MHCII^high^Ly6C^high^ interstitial macrophages within Ly6C^high^ interstitial macrophages and CD45^+^ leukocytes (Supplementary Figs. 5D and F). Furthermore, the average expression of MHCII protein in Ly6C^high^ interstitial macrophages was significantly increased after TAC in normal Se fed mice but not in the mice fed with high Se diet (Supplementary Fig. 5G). Histogram shows that the frequency distribution of MHCII^high^Ly6C^high^ interstitial macrophages was also significantly increased after TAC in the mice fed with normal Se but high Se diet abolished these changes (Supplementary Fig. 5H).

**Fig. 7:**
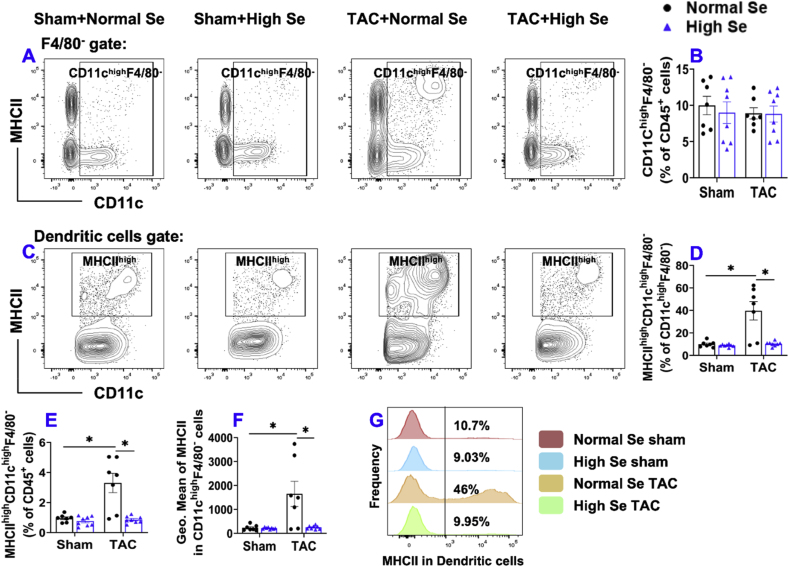
**High Se diet significantly attenuated chronic TAC-induced pulmonary dendritic cells (CD11c**^**high**^**F4/80**^**-**^**) activation.** (A) Flow cytometry plots of lung CD11c^high^F4/80^-^ cells. (B) Quantified data of the percentage of CD11c^high^F4/80^-^ cells within CD45^+^ cells. (C) Flow cytometry plots used for the detection of MHCII expression in CD11c^high^F4/80^-^ cells. (D, E) Quantified data of the percentages of MHCII^high^CD11c^high^F4/80^-^ cells within CD11c^high^F4/80^-^ cells and CD45^+^ cells. (F) Quantified data of mean fluorescent intensity of MHCII in CD11c^high^F4/80^-^ cells. (G) Representative histograms of MHCII expression in CD11c^high^F4/80^-^ cells of the indicated groups. *p < 0.05; Se, Selenium; n = 7 to 8 per group.

**Fig. 8:**
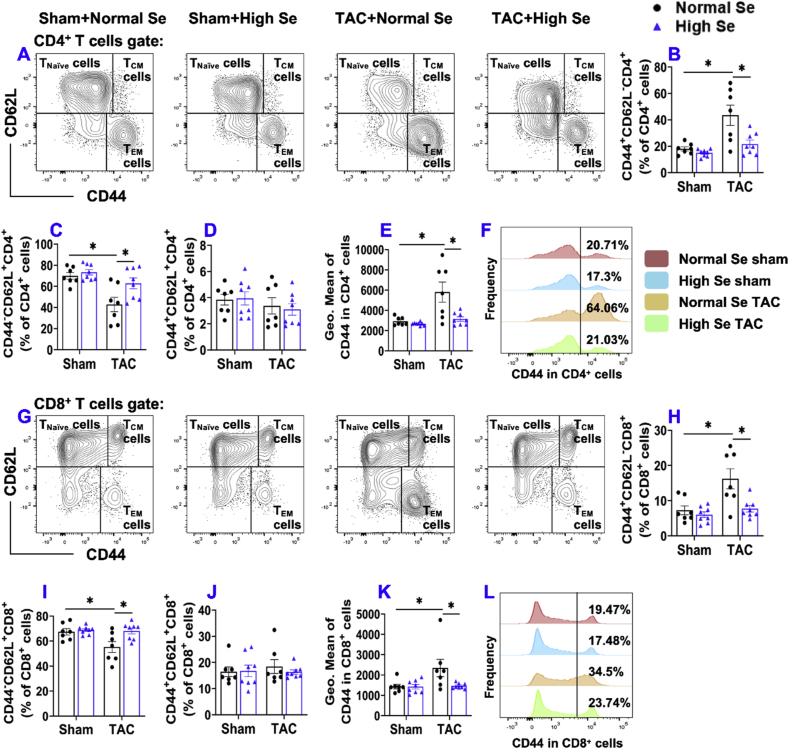
**High Se diet significantly attenuated chronic TAC-induced pulmonary T cell activation.** (A) Flow cytometry plots used for the detection of activation status of CD4^+^ T cells. (B–D) Quantified data of the percentages of CD44^+^CD62L^−^CD4^+^ effector memory cells, CD44^−^CD62L^+^CD4^+^ naïve cells, and CD44^+^CD62L^+^CD4^+^ central memory cells within CD4^+^ T cells, respectively. (E) Quantified data of mean fluorescent intensity of CD44 in CD4^+^ T cells. (F) Representative histograms of CD44 expression in CD4^+^ T cells of the indicated groups. (G) Flow cytometry plots used for the detection of activation status of CD8^+^ T cells. (H–J) Quantified data of the percentages of CD44^+^CD62L^−^CD8^+^ effector memory cells, CD44^−^CD62L^+^CD8^+^ naïve cells, and CD44^+^CD62L^+^CD8^+^ central memory cells within CD8^+^ T cells, respectively. (K) Quantified data of mean fluorescent intensity of CD44 in CD8^+^ T cells. (L) Representative histograms of CD44 expression in CD8^+^ T cells of the indicated groups. *p < 0.05; Se, Selenium; T_CM_ cells, Central Memory T cells; T_EM_ cells, Effector Memory T cells; n = 7 to 8 per group.

7. **High Se diet significantly attenuated chronic TAC-induced pulmonary CD11c**^+^ **dendritic cell activation.** Previous studies from others showed that Se plays a key role in regulating the immune system partially through modulating antigen-presenting cell proliferation and activation [[45], [46], [47]]. Since our previous study demonstrated that CD11c^+^ dendritic cells, a subset of professional antigen presenting cells, play an important role in TAC-induced cardiac hypertrophy and dysfunction [16], we further determined pulmonary dendritic cells in both sham or TAC conditions (Fig. 7). To avoid the influence of pulmonary CD11c^+^ alveolar macrophages, pulmonary dendritic cells were identified as F4/80^−^CD11c^+^ cells as shown in the gating strategy (Supplementary Fig. 1). There were no significant differences in the percentages of dendritic cells within CD45^+^ leukocytes in sham or TAC mice with or without high Se diet (Fig. 7A and B). The percentages of MHCII^high^ dendritic cells within dendritic cells or CD45^+^ leukocytes were significantly increased after TAC in the mice fed with normal Se, while these changes were attenuated by high Se diet (Fig. 7C, D, E). The average expression of MHCII protein in dendritic cells as demonstrated by GEO mean was significantly increased after TAC in the mice fed with normal Se, while high Se diet attenuated TAC-induced increase in MHCII expression in dendritic cells (Fig. 7F). Histogram shows that the frequency of MHCII^high^ dendritic cells was significantly increased after TAC in the mice fed with normal Se, while high Se diet abolished these changes (Fig. 7G).

8. **High Se diet significantly attenuated chronic TAC-induced pulmonary T cell activation.** Se regulates immunological response at least partially through modulating T cell proliferation, differentiation, and activation [46,47]. Since we previously demonstrated that T cell activation plays an important role in TAC-induced HF development [3], we further determined pulmonary total T cell subset (CD3^+^ cells), helper T cell subset (CD4^+^ T cells), cytotoxic T cells (CD8^+^ T cells), and their activation status according to their expression of CD44 and CD62L. The percentages of CD3^+^, CD4^+^, and CD8^+^ cells within CD45^+^ leukocytes were significantly decreased after TAC in the mice fed with normal Se, but not in those mice fed with high Se (Supplementary Figs. 6B, D, E). High Se diet significantly attenuated TAC-induced increase of CD3^+^ effector memory T cells (CD44^+^CD62L^−^CD3^+^) and decrease of CD3^+^ naïve T cells (CD44^−^CD62L^+^CD3^+^) (Supplementary Figs. 7B and C). The percentages CD3^+^ central memory T cells (CD44^+^CD62L^+^CD3^+^) were similar in the different groups (Supplementary Fig. 7D). High Se diet significantly attenuated the TAC-induced increase of CD44 (a protein facilitates immune cell tissue homing) expression in CD3^+^ T cells (Supplementary Fig. 7E). In addition, the histogram shows that the frequency of CD44^+^CD3^+^ T cells was significantly increased after TAC in the mice fed with normal Se, while the high Se diet abolished the above change (Supplementary Fig. 7F).
CD3^+^ T cells were further grouped into CD4^+^ and CD8^+^ T cells. As anticipated, TAC caused significant increase in effector memory CD4^+^ T cells (CD44^+^CD62L^−^CD4^+^) and significant decrease in naïve CD4^+^ T cells (CD44^−^CD62L^+^CD4^+^) in the mice fed with normal Se diet (Fig. 8A–C). TAC-induced increase of effector memory CD4^+^ T cells and decrease of naïve CD4^+^ T cells were abolished with high Se diet (Fig. 8A–C). The percentages of central memory CD4^+^ T cells (CD44^+^CD62L^+^CD4^+^) were unaffected by TAC or Se diets (Fig. 8D). High Se diet abolished TAC-induced increase in expression of CD44 in CD4^+^ T cells (Fig. 8E). Histogram shows that the frequency of CD44^+^CD4^+^ T cells was significantly increased after TAC in the mice fed with normal Se, while high Se diet abolished above change (Fig. 8F). Moreover, high Se diet significantly attenuated TAC-induced increase of effector memory CD8^+^ T cells (CD44^+^CD62L^−^CD8^+^) and significant decrease of naïve CD8^+^ T cells (CD44^−^CD62L^+^CD8^+^) (Fig. 8G–I). The percentages of central memory CD8^+^ T cells (CD44^+^CD62L^+^CD8^+^) were unchanged (Fig. 8J). The average expression of CD44 was significantly increased after TAC in the mice fed with normal Se diet but not in mice with high Se diet (Fig. 8K). Histogram shows that the frequency distribution of CD44 in CD8^+^ T cells was significantly increased after TAC in the mice fed with normal Se and high Se diet abolished these changes (Fig. 8L).

**Fig. 9:**
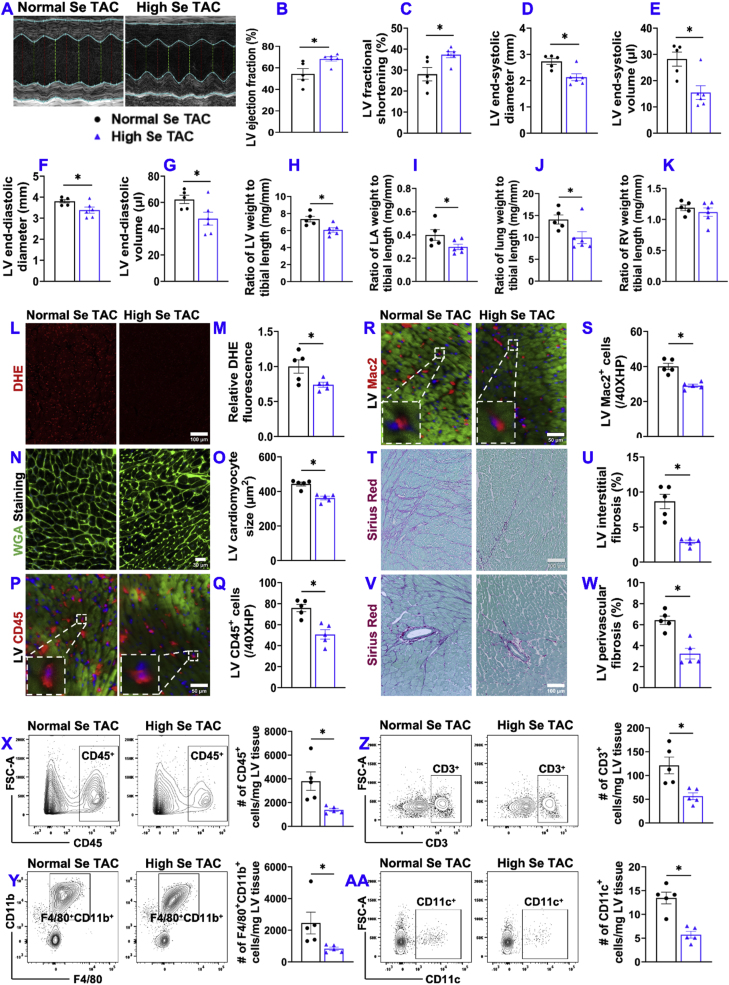
**High Se diet significantly attenuated TAC-induced early-phase cardiac oxidative stress, inflammation, cardiomyocyte hypertrophy, fibrosis, and dysfunction.** (A) Representative M-mode echocardiography images of the indicated groups. (B–G) Quantified data of LV ejection fraction, LV fractional shortening, LV end-systolic diameter, LV end-systolic volume, LV end-diastolic diameter, and LV end-diastolic volume. (H–K) The ratio of LV weight, LA weight, lung weight, and RV weight to tibial length. (L, M) Representative images of dihydroethidium (DHE) staining and quantified data of DHE staining. (N, O) Representative images of wheat germ agglutinin (WGA) staining and quantified LV cardiomyocyte cross-sectional area of the indicated groups. (P–S) Representative images and quantified data of LV CD45^+^ leukocytes and Mac2^+^ leukocytes infiltration performed by immuno-histological staining. (T–W) Representative images and quantified data of LV interstitial and perivascular fibrosis based on Sirius red/Fast green staining of the indicated groups. (X-AA) Representative flow cytometry plots and quantified data of numbers of CD45^+^ leukocytes, F4/80^+^/CD11b^+^ macrophages, CD3^+^ T cells, and CD11c^+^ dendritic cells per mg of LV tissue. *p < 0.05; Se, Selenium; n = 5 to 6 per group. *p < 0.05; Se, Selenium; n = 5 to 6 per group. (For interpretation of the references to colour in this figure legend, the reader is referred to the Web version of this article.)
9. **High Se diet also significantly attenuated TAC-induced early-phase LV oxidative stress, inflammation, cardiomyocyte hypertrophy, fibrosis, and dysfunction in mice.** Since TAC-induced cardiac immune cell infiltration generally peaks ∼7 days after TAC, we further determined the effect of high Se diet on TAC-induced LV inflammation and oxidative stress in mice one week after TAC. Interestingly, high Se diet also significantly attenuated TAC-induced LV dysfunction as compared with mice fed by normal Se diet (Fig. 9A–C). The TAC-induced increases in LV end-systolic diameter, end-systolic volume, end-diastolic diameter, and end-diastolic volume were also significantly attenuated in the mice fed with high Se diet (Fig. 9D–G). In addition, high Se diet significantly attenuated TAC-induced increases of LV weight, LA weight, lung weight, and their ratios to bodyweight or tibial length (Fig. 9H–K and Supplementary Table 3). Furthermore, high Se diet significantly attenuated TAC-induced LV oxidative stress (Fig. 9L and M), and LV cardiomyocyte hypertrophy (Fig. 9N and O), LV CD45^+^ leukocyte infiltration (Fig. 9P and Q), LV Mac2^+^ leukocyte infiltration (Fig. 9R and S), and LV fibrosis (Fig. 9T–W). Consistent with the reduced cardiac immune cell infiltration after high Se diet in mice, TAC-induced cardiac CD45^+^ leukocytes, F4/80^+^/CD11b^+^ macrophages, CD3^+^ T cells, and CD11c^+^ dendritic cells per mg of LV tissue and/or per LV were also significantly attenuated by high Se diet (Fig. 9X–AA and Supplementary Fig. 9).

## 4 Discussion

In the present study, we found that high Se diet significantly attenuated TAC-induced LV hypertrophy and dysfunction. We further demonstrated that high Se diet significantly ameliorated TAC-induced LV expression of 3-nitrotyrosine and 4-HNE, and LV leukocyte infiltration, indicating that high Se diet attenuated TAC-induced LV inflammation and oxidative stress. In addition, we found that high Se diet reduced TAC-induced pulmonary oxidative stress as evidenced by reduced pulmonary 3-nitrotyrosine and 4-HNE. Moreover, high Se diet significantly attenuated TAC-induced increases of lung weight, pulmonary leukocyte infiltration, fibrosis, vessel muscularization. Furthermore, high Se diet attenuated TAC-induced accumulation and activation of pulmonary macrophages, CD11c^+^ dendritic cells, CD4^+^ T cells, and CD8^+^ T cells. In addition, high Se diet significantly attenuated TAC-induced LV dysfunction, oxidative stress, cardiomyocyte hypertrophy, inflammation, and fibrosis at 7 days after TAC. These data suggest that Se plays a major role in systolic overload-induced HF using a model with normal Se at baseline.

One of the major findings of the present study is that high Se diet significantly attenuated TAC-induced HF development, evidenced by following changes. First, high Se diet attenuated TAC-induced decrease of LV ejection fraction, increase of LV end-diastolic diameter, and increase of LV end-systolic diameter. In addition, high Se diet significantly suppressed TAC-induced increases of lung weight and its ratio to tibial length, two commonly used objective markers for severe LV dysfunction or HF [6,20,39,42,48]. Moreover, high dietary Se ameliorated TAC-induced pulmonary inflammation, fibrosis, and vessel remodeling [2,6,7]. Our findings are conceptually consistent with previous clinical reports that dietary Se plays an important role in treating HF and impaired exercise tolerance [9,31,32], cardiomyopathy [33,34], and in attenuating inflammatory response after Dextran Sulfate Sodium-induced colitis [37].

High Se diet attenuated TAC-induced HF development likely through a collective effect on reducing TAC-induced LV oxidative stress, inflammation, hypertrophy, and fibrosis. Indeed, we found that high Se diet significantly reduced TAC-induced LV hypertrophy or cardiomyocyte hypertrophy. The expressions of 3-nitrotyrosine and 4-HNE are commonly used as oxidative stress markers, and it is well established that oxidative stress is increased and contributes to HF development [19,20,22,42,48]. The findings that high Se diet effectively attenuated TAC-induced cardiac 3-nitrotyrosine and 4-HNE expressions indicate that reduced LV oxidative stress contributed to the cardiac protective effect by high Se diet. In addition, high Se diet also significantly attenuated TAC-induced LV leukocyte infiltration or inflammation. Although LV hypertrophy, oxidative stress, inflammation, and fibrosis can all independently promote LV dysfunction or HF development [8,19,22,42], since oxidative stress and inflammation can cause cardiomyocyte and vessel injury, cardiomyocyte hypertrophy, and induction of fibrosis by promoting the transition from fibroblast to myofibroblast [3,8,15,16,42,49], the reduced LV oxidative stress and inflammation by high Se diet might play central roles in attenuating HF development. Although it is generally believed that Se supplements attenuate oxidative stress mainly through the increase of the production of various selenoproteins, however, Se supplements also suppress mitochondrial ROS production and improve mitochondrial respiration in cultured human cardiomyocytes [9], suggesting that high Se diet might also exert its protection through improving cardiomyocyte metabolism and survival capacity [9].

In addition to the cardiac protective effect, the protective effects of high Se diet against TAC-induced HF progression may be partially through impacting oxidative stress and inflammation in pulmonary tissues. As we previously reported, the increase in LV after-load caused by TAC leads to pathologic LV hypertrophy, a decrease in LV ejection fraction, and an increase in LV end-diastolic pressure [6]. The increased LV filling pressure resulted in LA hypertrophy, an increase in pulmonary venous pressure, and consequent pulmonary inflammation. The increased pulmonary venous pressure and pulmonary inflammation will further promote pulmonary inflammation, vessel remodeling, and pulmonary artery pressure. Heart failure-induced pulmonary artery hypertension will further cause RV hypertrophy, RV dysfunction, and increased RV end-diastolic pressure and RA hypertrophy [6]. LV failure induced pulmonary remodeling and RV hypertrophy are modulated by inflammation and oxidative stress as enhance of lung inflammation by air pollution promotes pulmonary remodeling and RV hypertrophy [11], while attenuating inflammation and oxidative stress can attenuate TAC-induced pulmonary inflammation and remodeling and RV hypertrophy in mice with preexisting LV failure [7,23]. The TAC-induced increases of pulmonary 3-nitrotyrosine and 4-HNE in the present study are consistent with our previous reports [11,12,23], and the reduced pulmonary oxidative stress likely contributed to the reduced pulmonary inflammation and remodeling in these mice. In the context that inflammation plays an important role in TAC-induced HF development and progression [3,6,7,11,16,20], the significantly reduced pulmonary oxidative stress and leukocyte infiltration in mice after high Se diet indicate that high Se diet likely exerted the protection partially through modulating lung remodeling.

GPX1 and GPX4 are important selenoproteins that scavenge hydrogen peroxide and reduce oxidative stress in various tissues [9,40,[50], [51], [52], [53], [54], [55]]. Interestingly, TAC significantly increased LV GPX1 and GPX4 protein expressions in normal Se-fed mice. The increased cardiac GPX1 and GPX4 protein expressions in these mice were likely a compensatory response against the increased oxidative stress in LV tissue, but the increased cardiac GPX1 and GPX4 expressions were clearly insufficient to control the increased oxidative stress. One possible explanation is that oxidative stress in response to pressure overload arises from various sources such as mitochondrial-derived ROS, increases of NADPH oxidase (an enzyme highly expressed in the infiltrated macrophages) [21,56], and xanthine oxidase [18], uncoupled NO synthase [39,42,48], and decreased antioxidant expression (such as SOD3) [20,22], etc. Thus, the increased GPX1 and GPX4 protein expressions were unable to overcome the drastically increased oxidative stress from diverse cells and different cellular compartments.

Dendritic cells, macrophages, and T cells are increased in maladaptive LV hypertrophy and HF development [3,7,13,16]. We previously found that both activated CD4^+^ T cells (CD44^+^CD62L^−^CD4^+^ T cells), CD8^+^ T cells (CD44^+^CD62L^−^CD8^+^ T cells), and CD11c^+^ APCs (particularly the MHCII^+^CD11c^+^ APCs) were increased in LV tissues in HF mice [3,16], and inhibition of the crosstalk between T cells and APCs by CD28 knockout, CD86/CD80 double knockout, or depletion of CD11c^+^ APCs significantly attenuated TAC-induced LV infiltration of the activated CD4^+^ T cells, CD8^+^ T cells, MHCII^+^CD11c^+^ APCs, and LV inflammation and HF development [3,16]. Our studies further demonstrated that TAC-induced HF was associated with increased pulmonary activated APCs, activated T cells, macrophages, NK cells, and neutrophils [14,17]. Here, we found that high Se diet significantly attenuated TAC-induced pulmonary accumulation of activated CD44^+^CD62L^−^CD4^+^ T cells, CD44^+^CD62L^−^CD8^+^ T cells, MHCII^+^CD11c^+^ APCs, and activated macrophages. These findings indicate that the Se diet suppressed TAC-induced pulmonary activation and infiltration of APCs and T cells in these mice. Previous studies have demonstrated that Se supplement attenuated viral infection and/or cancer growth by enhancing the proliferation and activation of CD4^+^ T cells, CD8^+^ T cells, natural killer cells, and/or APCs [35]. Our findings that high Se diet suppressed rather than enhanced TAC-induced activation of pulmonary T cells and APCs indicate that Se effect on the activation of T cells and APCs is partially dependent on the initial tissue Se status and the disease conditions. We postulate that mice fed with normal Se diet still had sufficient Se to maintain the required activation of T cells and APCs after TAC, and the increased pulmonary accumulation and activation of T cells and APCs were predominantly a pathological response to the TAC-induced cardiac and pulmonary injury in these mice. Inhibitions of chemokines and their corresponding receptors, such as inhibition of the CXCL1-CXCR2 axis and CCL2-CCR2 axis, have been suggested as a therapeutic approach in treating cardiovascular diseases such as HF, coronary artery diseases, and cardiac arrhythmia [[57], [58], [59], [60], [61]]. For example, previous studies demonstrated that the CXCL1-CXCR2 axis and CCL2 (MCP-1)-CCR2 axis play important roles in promoting TAC or angiotensin-II induced cardiac inflammation, hypertrophy, fibrosis, and dysfunction [57,60,62], while inhibition of CXCL1-CXCR2 or CCL2-CCR2 genetically or pharmacologically were effective in attenuating cardiac immune cell infiltration, hypertrophy, and dysfunction [57,60,62]. Our previous studies also demonstrated that TAC-induced HF is associated with increased cardiac and pulmonary CCL2 (MCP-1) mRNA and/or protein expression [6,7,12,14,17,23]. Furthermore, studies also showed that selenium supplements were effective in attenuating oxidative stress, macrophage infiltration, and/or MCP-1 expression in diseases such as cardiomyopathy, cardiac hypertrophy, colitis, and leukemia [36,37,63,64]. Since the interactions between chemokines and their corresponding receptors play important roles in facilitating systolic overload-induced cardiac and pulmonary immune cell infiltration and migration, and since systolic overload enhanced the CXCL1-CXCR2 axis and CCL2-CCR2 axis [6,14,17,57,60,62], the TAC-induced cardiopulmonary immune cell infiltration is likely also partially modulated by CXCL1-CXCR2 and CCL2-CCR2 signaling.

This study has several limitations. First, high Se diet attenuated pressure overload-induced LV oxidative stress, leukocyte infiltration, fibrosis, and cardiomyocyte hypertrophy. Since all these factors could independently regulate LV dysfunction, the present study could not fully differentiate the relative contribution of each of these factors. Second, the present study only investigated the role of high Se diet on TAC-induced HF development in male mice. Since female mice are resistant TAC-induced LV hypertrophy and dysfunction, the effect of high Se diet on TAC-induced oxidative stress, inflammation, and dysfunction may be different between male and female mice. However, as HF development and HF-induced pulmonary remodeling are commonly observed in both male and female mice, and since oxidative stress and immune responses occur in both genders in human and in mice, high Se diet likely also exerts a protective effect against TAC-induced cardiac oxidative stress, inflammation, and HF development in female mice.

In summary, we demonstrated the protective effect of high dietary Se on pressure overload-induced HF development and progression in WT male mice. High Se diet significantly attenuated pressure overload-induced cardiac dysfunction, LV hypertrophy, cardiopulmonary inflammation, oxidative stress, lung remodeling (arteriole muscularization and fibrosis), and RV hypertrophy. The TAC-induced accumulation of macrophages and activation of dendritic cells, macrophages, and T cells in the lungs were also significantly attenuated with high Se diet. Therefore, dietary high Se diet may represent an attractive therapeutic strategy for the treatment of pressure overload-induced HF in patients.

## Funding

The research activity was supported by 10.13039/100000002NIH research grants R01HL161085, R01HL139797, P20GM104357, and P30GM149404.

## CRediT authorship contribution statement

**Umesh Bhattarai:** Writing – review & editing, Writing – original draft, Methodology, Investigation, Formal analysis, Conceptualization. **Rui Xu:** Methodology, Investigation, Formal analysis, Conceptualization. **Xiaochen He:** Methodology, Investigation, Formal analysis, Conceptualization. **Lihong Pan:** Methodology, Formal analysis. **Ziru Niu:** Methodology, Formal analysis. **Dongzhi Wang:** Methodology, Formal analysis. **Heng Zeng:** Writing – review & editing. **Jian-Xiong Chen:** Writing – review & editing. **John S. Clemmer:** Writing – review & editing. **Yingjie Chen:** Writing – review & editing, Writing – original draft, Validation, Supervision, Methodology, Investigation, Funding acquisition, Formal analysis, Conceptualization.

## Declaration of competing interest

The authors have no conflicts of interest to disclose.

## Data Availability

Data will be made available on request.
