# Supplementary material for: High selenium diet attenuates pressure overload-induced cardiopulmonary oxidative stress, inflammation, and heart failure
Source: Redox Biol. 2024 Aug 22;76:103325. doi: 10.1016/j.redox.2024.103325 (PMC11399737; doi:10.1016/j.redox.2024.103325)
Supplement: Multimedia component 1 [file mmc1.docx]

**Supplementary materials**

**High selenium diet attenuates pressure overload-induced cardiopulmonary oxidative stress, inflammation, and heart failure**

Umesh Bhattarai^a^*****, Rui Xu^a^*****, Xiaochen He^a^, Lihong Pan^a^, Ziru Niu^a^, Dongzhi Wang^a^, Heng Zeng^b^, Jian-Xiong Chen^b^, John S. Clemmer^a^, Yingjie Chen^a^

^a^Department of Physiology and Biophysics, School of Medicine, University of Mississippi Medical Center, Jackson, MS, United States

^b^Department of Pharmacology and Toxicology, School of Medicine, University of Mississippi Medical Center, Jackson, MS, United States

*These authors contribute equally to this study.

**Correspondence**

Yingjie Chen, Ph.D.

Professor

Department of Physiology and Biophysics,

University of Mississippi Medical Center,

2500 North State Street,

Jackson, MS, 39216

Office: 601-815-3986

Email: [ychen2@umc.edu](mailto:ychen2@umc.edu)

**Animals and Experimental protocols:** Male C57BL/6J mice were purchased from Jackson Laboratory (Bar Harbor, ME) and were housed in ventilated cages in a temperature-controlled environment with 12-hour light/dark cycles. The mice were randomly divided into two groups and fed with standard chow diet containing normal Se diet (80 ppb Se) or high Se diet (400 ppb Se) (Envigo RMS Holding Corp.) for the rest of the experimental duration. One week after normal or high Se diets, these mice were subjected to either sham surgery or TAC, a commonly used surgical procedure to generate systolic overload to mimic clinical conditions such as aortic stenosis or hypertension. The TAC surgery was performed in mice using a 26-guage blunt needle after anesthesia with intraperitoneal injection of Ketamine (100 mg/Kg) and xylazine (10 mg/Kg) (1, 2). Final cardiac functional test was performed eight weeks after the surgery. After the cardiac functional test, the cardiac and pulmonary samples were collected and used for further histological, immune-histological, and biochemical analyses. Mice fed with normal or high Se diets were also subjected to TAC for one week to determine the early-phase effect of Se on TAC-induced cardiac dysfunction, oxidative stress, inflammation, and fibrosis. The experimental studies were approved by the Institutional Animal Care and Use Committee at the University of Mississippi Medical Center.

**Echocardiography:** Echocardiography was performed using a VisualSonics Vevo 2100 imaging system (FUJIFILM VisualSonics Inc., Canada) as previously described (2). During the recording procedure, the mice were anesthetized by inhalation of 1-2% isoflurane mixed with 100% oxygen. M-mode echocardiographic recordings were analyzed using Vevo LAB software (FUJIFILM VisualSonics Inc., Canada) to measure heart rate, LV ejection fraction, LV fractional shortening, LV end-systolic diameter, LV end-diastolic diameter, LV end-systolic volume, LV end-diastolic volume, LV anterior and posterior wall thickness at end-systole or end-diastole, stroke volume, and cardiac output.

**Histological and Immune-histological Staining:** The heart and lung tissue sections were deparaffinized and rehydrated. Lung fibrosis was performed using Masson’s trichrome staining kit (ThermoFisher Scientific). Cardiac fibrosis was performed using Sirius Red/Fast Green Staining Kit (Chondrex Inc.). FITC-conjugated wheat germ agglutinin (WGA) staining was used to measure LV cardiomyocyte cross-sectional area. Dihydroethidium (DHE) staining was performed to determine relative ROS production in the cardiac and pulmonary tissues. In brief, the cardiac and pulmonary tissue sections were incubated with DHE (D23107, Invitrogen, 10µM in PBS) in a light-protected humidified chamber at 37°C for 30 minutes, washed with PBS, and mounted with 4',6-diamidino-2-phenylindole (DAPI) containing mounting media (enQuire BioReagents, QS4-20ML) to visualize the nucleus. Tissue fibrosis, LV cardiomyocyte size, and relative DHE intensity were quantified using ImageJ software from the National Institute of Health. The infiltrated CD45^+^ and Mac2^+^ leukocytes in the LV and lung tissues were stained with goat anti-CD45 antibody (R&D Systems, AF114, 1;100 dilution) and goat anti-Mac2 antibody (R&D Systems, AF1197, 1:150 dilution), respectively. The CD45^+^ and Mac2^+^ leukocytes were visualized using Alexa Flour-555 conjugated donkey anti-goat secondary antibody (Invitrogen, 1:1000 dilution). Muscularization of pulmonary arterioles was determined by using mouse anti-α smooth muscle actin (αSMA) (Invitrogen, 14-9760-82, 1:200 dilution) and rabbit anti-CD31 antibody (Cell Signaling Technologies Inc., 77699, 1:200 dilution). The αSMA and CD31 were then visualized by using Alexa Flour-555 conjugated goat anti-mouse (Invitrogen, 1:1000 dilution) and Alexa Flour-594 conjugated goat anti-rabbit (Invitrogen, 1:1000 dilution) secondary antibodies, respectively. The tissue sections were then mounted with 4',6-diamidino-2-phenylindole (DAPI) containing mounting media (enQuire BioReagents, QS4-20ML) to visualize the nucleus. The histological images were captured using Mantra Quantitative Pathology Imaging System (Perkin Elmer), and infiltrated CD45^+^ and Mac2^+^ leukocytes were quantified using inForm software version 2.2.1 (Perkin Elmer).

**Western Blot analyses:** The western blot analyses were performed as previously described (3). In brief, LV and lung tissues were homogenized in RIPA lysis buffer (ThermoFisher Scientific, 89900) supplemented with 1X protease and phosphatase inhibitor (ThermoFisher Scientific, 1861280). The protein content of the tissue lysate was quantified using BCA protein assay kit (ThermoFisher Scientific, 23225). Then, 50µg tissue lysate was loaded, separated by SDS-PAGE, and transferred into methanol-activated PVDF membranes at 90 mV for 100 minutes. The membranes were then blocked with 5% non-fat milk in TBST and incubated with primary antibodies for atrial natriuretic peptide (ThermoFisher Scientific, PA5-29559, 1:1000 dilution), 3-nitrotyrosine (Abcam, ab61392, 1:1000 dilution), 4-hydroxynonenal (Abcam, ab48506, 1:1000 dilution), glutathione peroxidase 1 (R&D Systems, AF3798, 1:1000 dilution), glutathione peroxidase 4 (R&D Systems, MAB5437, 1:1000 dilution), and β-tubulin (Cell Signaling Technologies Inc., 2146, 1:1000 dilution) overnight at 4°C in a rocker. The membranes were then incubated with HRP-conjugated secondary antibodies (Abcam, ab97030, ab97064, ab97110, 1:4000 dilution in 5% non-fat dry milk in TBST) at room temperature for 1 hour. The protein bands were detected using an iBright FL1500 instrument (ThermoFisher Scientific). The relative protein expression was then quantified by using NIH ImageJ software.

**Flow Cytometry analyses:** Lung samples were harvested after perfusing the tissue with cold PBS through the right ventricle, and the lung samples were digested in Hank’s balanced salt solution (Life Technologies Corporation) supplied with 1 mg/mL collagenase D (Roche Diagnostics, Germany) at 37°C for 30 minutes using a tissue dissociator (Miltenyi Biotec) as previously described (4). The cell suspension was then passed through a 100 µm cell strainer to remove debris. The red blood cells in filtered cell suspension were then lysed with 2 mL of RBC lysis buffer (Life Technologies Corporation). The cells were then stained with fixable viability dye (FVS440UV, BD Bioscience, CA) in PBS. The cells were then incubated with CD16/32 antibody (clone 2.4G2, Biolegend, CA) to block the non-specific binding of antibodies to FcRγ. The cells were then incubated with fluorescence-conjugated multi-staining antibodies (Supplementary Table 1). Data were acquired on a BD FACSymphony^TM^ A3 Cell Analyzer (BD Biosciences, CA) and analyzed by FlowJo-V10 (FlowJO, OR) software. The gating strategy used for lung flow cytometry analysis is shown in Supplementary Figure 1. For the flow cytometry of LV tissue, LV tissues were minced into small pieces and digested with Deoxyribonuclease I (6.67 µg/ml, Sigma-Aldrich) and Liberase^TM^ (125 µg/ml, Roche Diagnostics, Germany) at 37°C for 30 minutes using a tissue dissociator (Miltenyi Biotec). Staining procedure is same as described above for lung tissue. Total number of immune cells per LV and/or per mg LV tissue was estimated by determining weight of LV tissue used for flow cytometry, total LV weight, total cells present in digested tissue sample, and percentage of each immune cell types in stained total cells. The gating strategy used for LV flow cytometry analysis is shown in Supplementary Figure 8.

**References**

1. Hu P, Zhang D, Swenson L, Chakrabarti G, Abel ED, Litwin SE. Minimally invasive aortic banding in mice: effects of altered cardiomyocyte insulin signaling during pressure overload. Am J Physiol Heart Circ Physiol. 2003;285(3):H1261-9.

2. Zhang P, Xu X, Hu X, van Deel ED, Zhu G, Chen Y. Inducible nitric oxide synthase deficiency protects the heart from systolic overload-induced ventricular hypertrophy and congestive heart failure. Circ Res. 2007;100(7):1089-98.

3. Bhattarai U, He X, Xu R, Liu X, Pan L, Sun Y, et al. IL-12α deficiency attenuates pressure overload-induced cardiac inflammation, hypertrophy, dysfunction, and heart failure progression. Front Immunol. 2023;14:1105664.

4. Wang H, Hou L, Kwak D, Fassett J, Xu X, Chen A, et al. Increasing Regulatory T Cells With Interleukin-2 and Interleukin-2 Antibody Complexes Attenuates Lung Inflammation and Heart Failure Progression. Hypertension. 2016;68(1):114-22.

**Supplementary Table 1.** Antibodies used for flow cytometry analyses.

| **Antibody** | **Conjugate** | **Clone** | **Vendor** | **Catalog #** |
| --- | --- | --- | --- | --- |
| **CD3e** | BUV737 | 145-2C11 | BD Biosciences | 612771 |
| **CD4** | BUV496 | GK1.5 | BD Biosciences | 612952 |
| **CD8α** | BB790 | 53-6.7 | BD Biosciences | 624296 |
| **CD11b** | BV650 | M1/70 | BioLegend | 101259 |
| **CD11c** | BV711 | N418 | BioLegend | 117349 |
| **CD16/32** | - | 93 | BioLegend | 101302 |
| **CD19** | BUV395 | 1D3 | BD Biosciences | 563557 |
| **CD44** | FITC | IM-7 | BD Biosciences | 553133 |
| **CD45** | BUV805 | 30-F11 | BD Biosciences | 568336 |
| **CD62L** | AF700 | MEL-14 | BD Biosciences | 104418 |
| **F4/80** | BUV563 | T45-2342 | BD Biosciences | 749284 |
| **I-A/I-E (MHC-II)** | APC-Cy7 | M5/114.15.2 | BioLegend | 107628 |
| **Ly6C** | BV605 | AL-21 | BD Biosciences | 563011 |
| **Ly6G** | AF700 | 1A8 | BioLegend | 127622 |
| **NK 1.1** | APC | PK136 | eBioscience | 17-5941-82 |

**Supplementary Table 2.** Anatomic data of male mice under sham and TAC conditions, with or without Se supplementation eight weeks after TAC.

| **Parameters** | **Sham+Normal Se**  **(n=15)** | **Sham+High Se**  **(n=9)** | **TAC+Normal Se**  **(n=16)** | **TAC+High Se (n=13)** |
| --- | --- | --- | --- | --- |
| Body Weight (g) | 31.13 ±0.62 | 31.21 ±0.82 | 27.4±1.02***** | 30.75±0.85**^†^** |
| Tibial Length (mm) | 17.59±0.08 | 17.62±0.14 | 17.54±0.07 | 17.58±0.16 |
| LV Weight (mg) | 93.77±1.94 | 92.84±3.41 | 184.67±7.6***** | 136±5.56**^#†^** |
| LA Weight (mg) | 3.71±0.18 | 3.52±0.30 | 19.87±3.81***** | 4.99±0.53**^†^** |
| Lung Weight (mg) | 141.41±2.95 | 136.08±3.25 | 261.48±31.82***** | 147.12±4.3**^†^** |
| RV Weight (mg) | 21.56±0.61 | 21.73±0.74 | 30.99±2.57***** | 21.41±0.58**^†^** |
| RA Weight (mg) | 3.95±0.24 | 3.7±0.24 | 5.19±0.51***** | 3.96±0.16**^†^** |
| Total Heart Weight (mg) | 123±2.47 | 121.8±4.33 | 240.72±13.06***** | 166.37±6.31**^#†^** |
| LV weight/BW (mg/g) | 3.03±0.08 | 2.97±0.07 | 7.04±0.6***** | 4.47±0.24**^†^** |
| LA weight/BW (mg/g) | 0.12±0.01 | 0.11±0.01 | 0.79±0.17***** | 0.17±0.02**^†^** |
| Lung weight/BW (mg/g) | 4.55±0.09 | 4.37±0.09 | 10.4±1.72***** | 4.81±0.14**^†^** |
| RV weight/BW (mg/g) | 0.69±0.02 | 0.70±0.02 | 1.19±0.14***** | 0.7±0.02**^†^** |

Data are mean ± SEM. *p<0.05 compared with Normal Se Sham, #p<0.05 compared with High Se Sham, **^†^**p<0.05 compared with Normal Se TAC; Se, Selenium; BW, body weight.

**Supplementary Table 3.** Anatomic data of male mice TAC conditions, with or without Se supplementation one week after TAC.

| **Parameters** | **TAC+Normal Se**  **(n=5)** | **TAC+High Se (n=6)** |
| --- | --- | --- |
| Body Weight (g) | 21.94±0.64 | 23.38±0.67 |
| Tibial Length (mm) | 16.63±0.03 | 16.54±0.08 |
| LV Weight (mg) | 122±5.37 | 100.27±4.14* |
| LA Weight (mg) | 6.64±0.78 | 4.9±0.35* |
| Lung Weight (mg) | 233.9±16.95 | 164.17±22.18* |
| RV Weight (mg) | 19.74±0.77 | 18.47±1.13 |
| RA Weight (mg) | 3.54±0.36 | 3.32±0.33 |
| Total Heart Weight (mg) | 151.9±5.89 | 126.95±4.7* |
| LV weight/BW (mg/g) | 5.58±0.3 | 4.29±0.17* |
| LA weight/BW (mg/g) | 0.3±0.03 | 0.21±0.02* |
| Lung weight/BW (mg/g) | 10.78±1.11 | 7.04±0.96* |
| RV weight/BW (mg/g) | 0.9±0.06 | 0.79±0.05 |

Data are mean ± SEM. *p<0.05 compared with Normal Se TAC; Se, Selenium; BW, body weight.


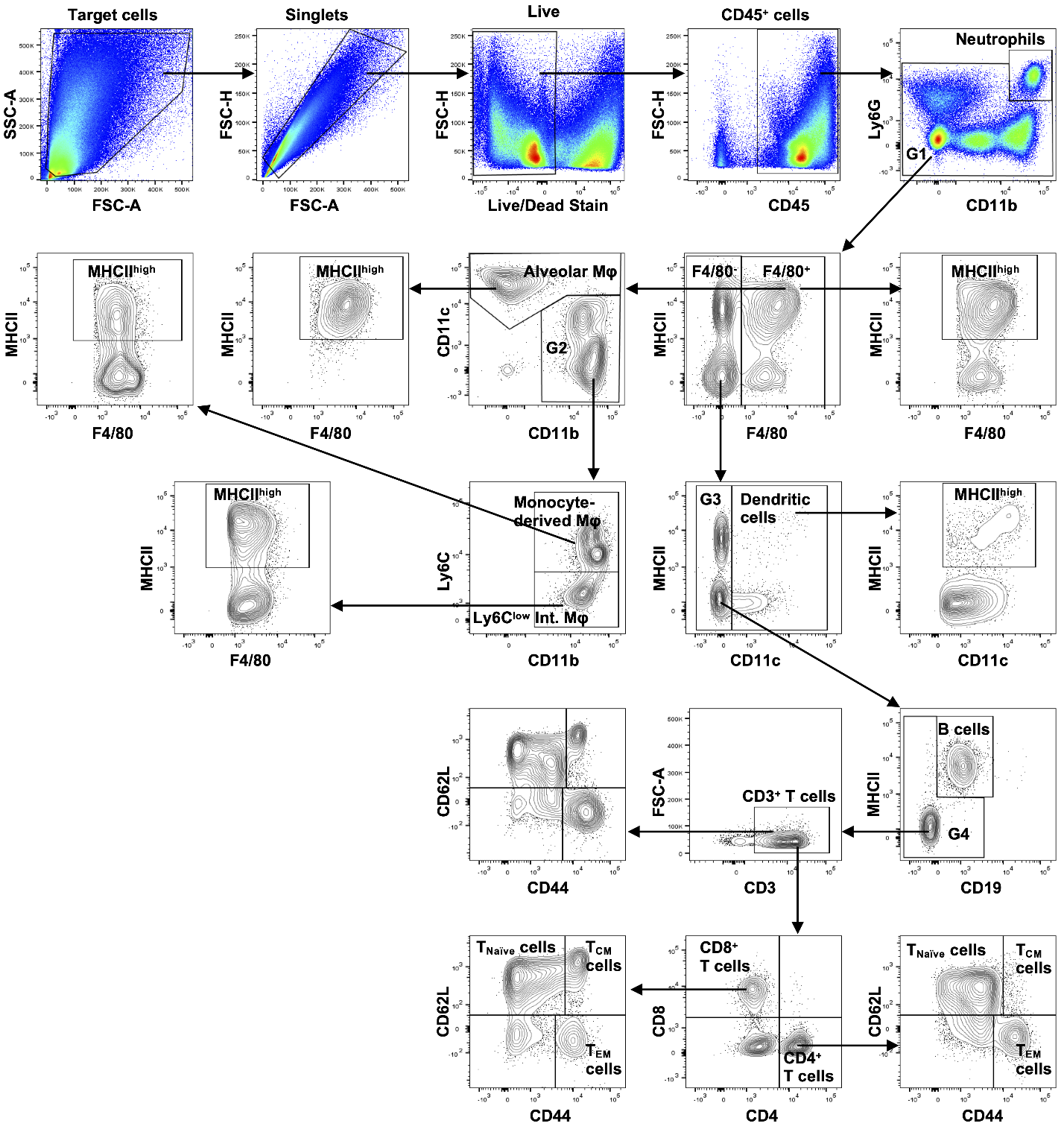


**Supplementary Figure 1.** Gating strategy used for the identification of Neutrophils, Macrophages, Dendritic cells, B cells, and T cells in the lung.


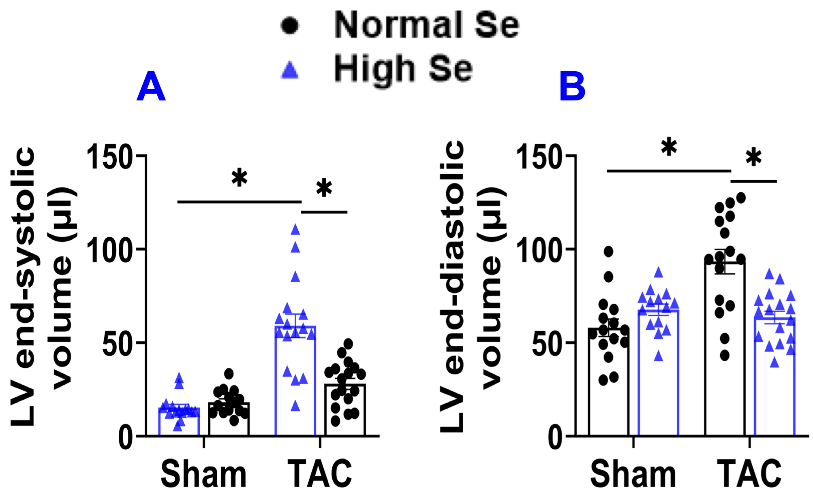


**Supplementary Figure 2.** (A, B) Quantified data of echocardiographic measurements of LV end-systolic volume, LV end-diastolic volume. *p<0.05; Se, Selenium; n=14 to17 per group.


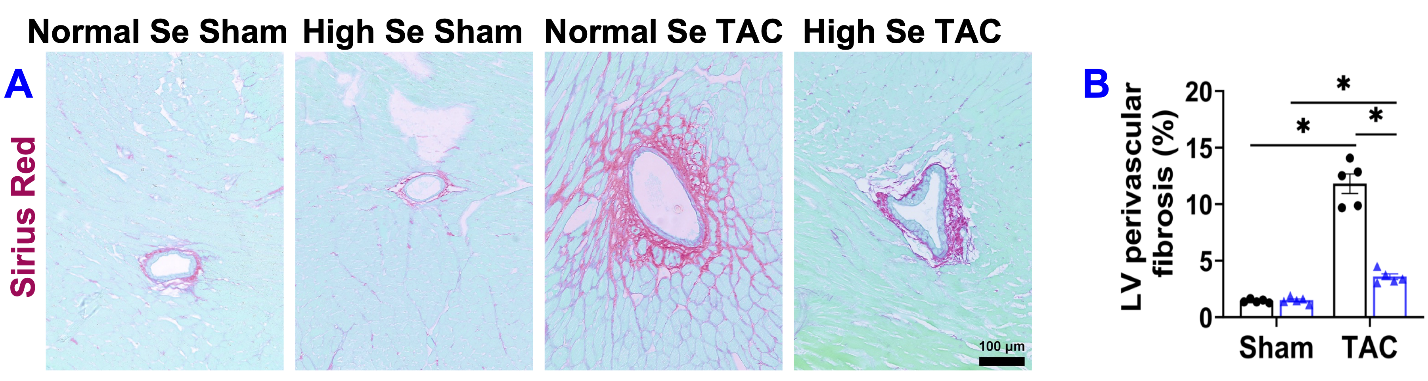


**Supplementary Figure 3.** (A, B) Representative images and quantified data of LV perivascular fibrosis performed by Sirius red/Fast green staining. *p<0.05; Se, Selenium; n=5 per group.

**
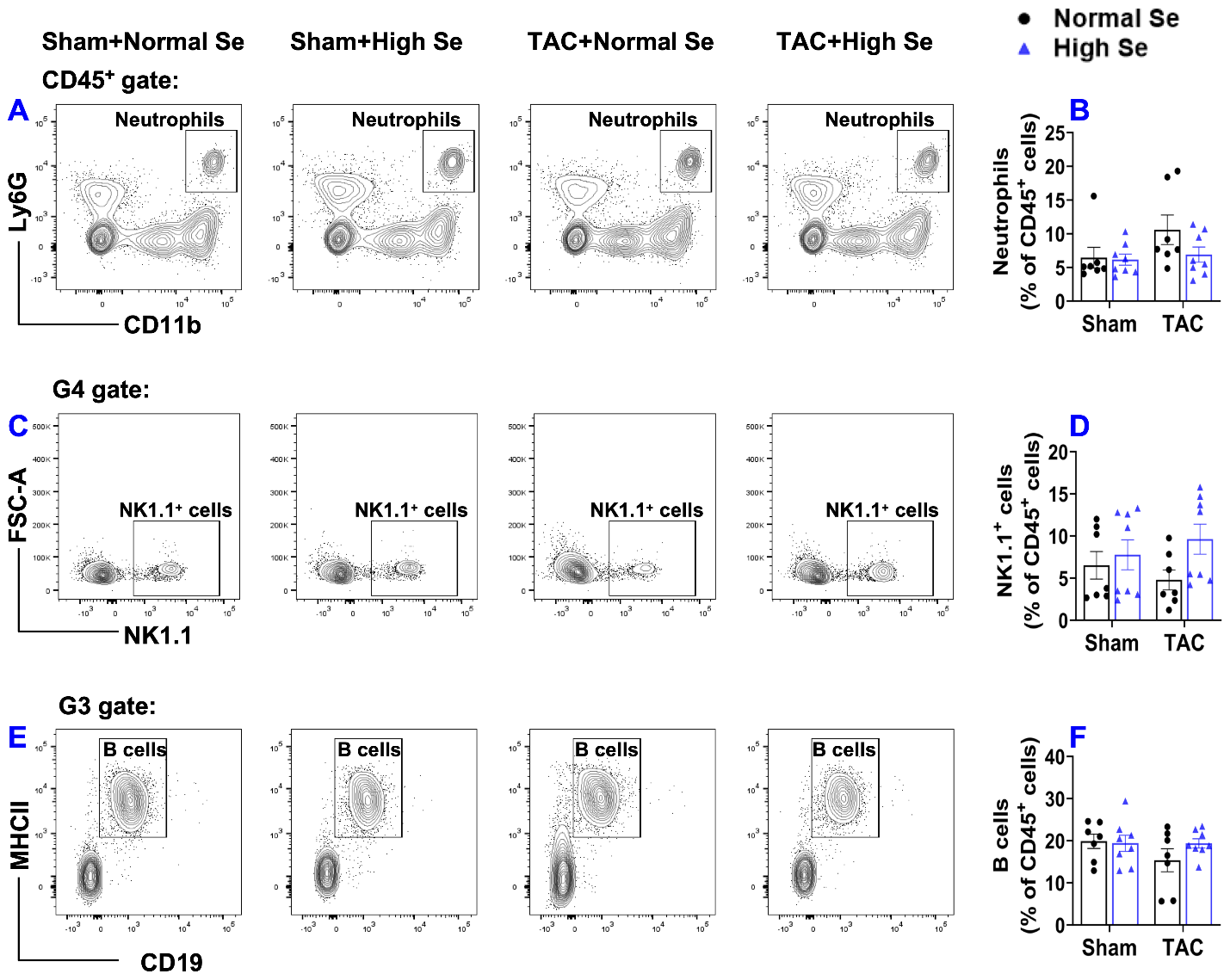
**

**Supplementary Figure 4.** Flow cytometry plots and quantified data of neutrophils, NK1.1^+^ cells, and B cells within CD45^+^ leukocytes. *p<0.05; Se, Selenium; n=7 to 8 per group.


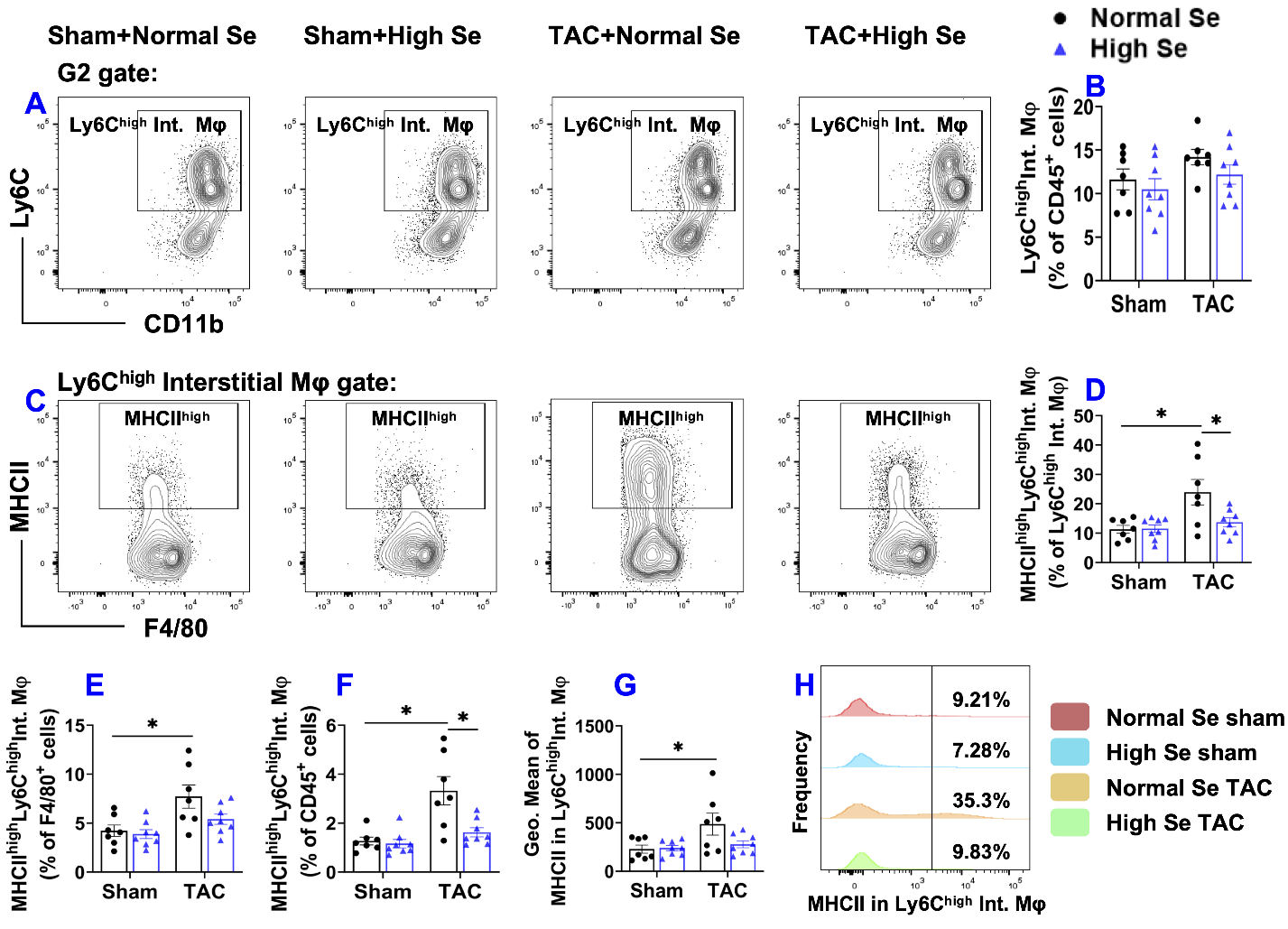


**Supplementary Figure 5.** High Se diet significantly attenuated chronic TAC-induced activation of monocyte-derived Ly6C^high^ interstitial macrophages (Ly6C^high^CD11c^low^CD11b^high^F4/80^+^). (A) Flow cytometry plots of lung Ly6C^high^ interstitial macrophages. (B) Quantified data of the percentage of Ly6C^high^ interstitial macrophages within CD45^+^ cells. (C) Flow cytometry plots used for the detection of MHCII expression in Ly6C^high^ interstitial macrophages. (D-F) Quantified data of the percentages of MHCII^high^Ly6C^high^ interstitial macrophages within Ly6C^high^ interstitial macrophages, F4/80^+^, and CD45^+^ cells, respectively. (G) Quantified data of mean fluorescent intensity of MHCII in Ly6C^high^ interstitial macrophages. (H) Representative histograms of MHCII expression in Ly6C^high^ interstitial macrophages of the indicated groups. *p<0.05; Se, Selenium; Mφ, macrophages; Int., interstitial; n=7 to 8 per group.


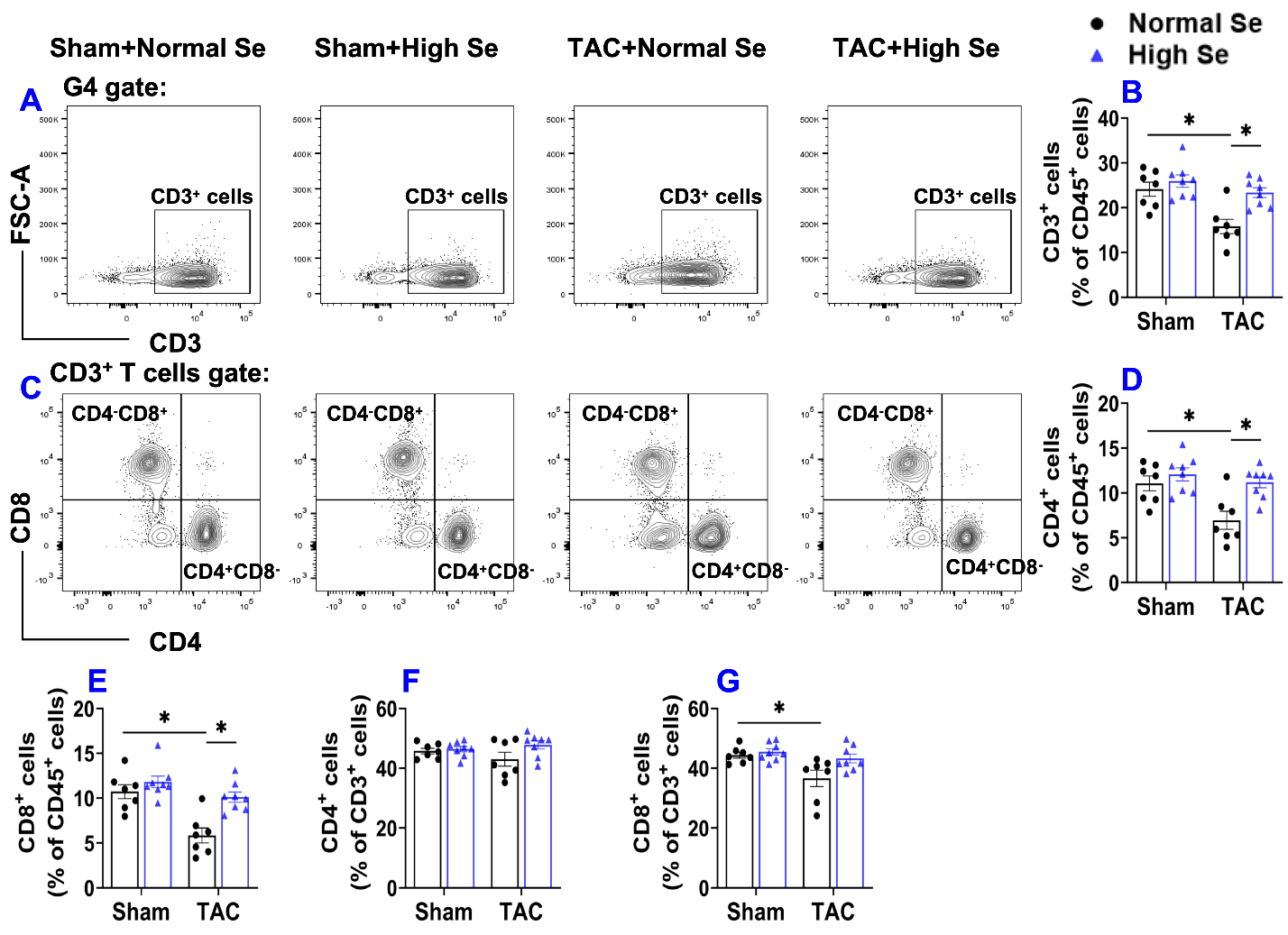


**Supplementary Figure 6.** Gating strategy used for the identification of CD3^+^, CD4^+^, and CD8^+^ T cells in the lung. *p<0.05; Se, Selenium; n=7 to 8 per group.


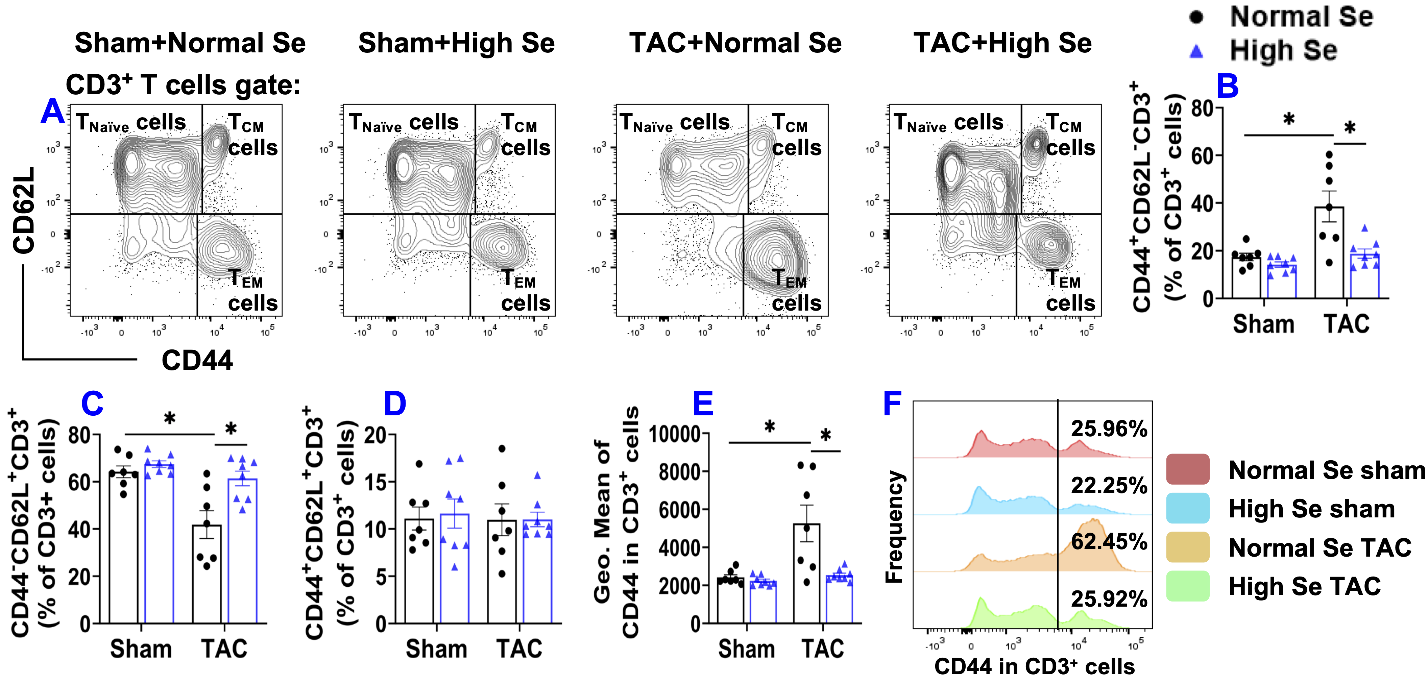


**Supplementary Figure 7.** (A) Flow cytometry plots used for the detection of activation status of CD3^+^ T cells. (B-D) Quantified data of the percentages of CD44^+^CD62L^-^ effector memory T cells, CD44^-^CD62L^+^ naïve T cells, and CD44^+^CD62L^+^ central memory T cells within CD3^+^ T cells, respectively. (E) Quantified data of mean fluorescent intensity of CD44 in CD3^+^ T cells. (F) Representative histograms of CD44 expression in CD3^+^ T cells of the indicated groups. *p<0.05; Se, Selenium; T_CM_ cells, Central Memory T cells; T_EM_ cells, Effector Memory T cells; n=7 to 8 per group.

**
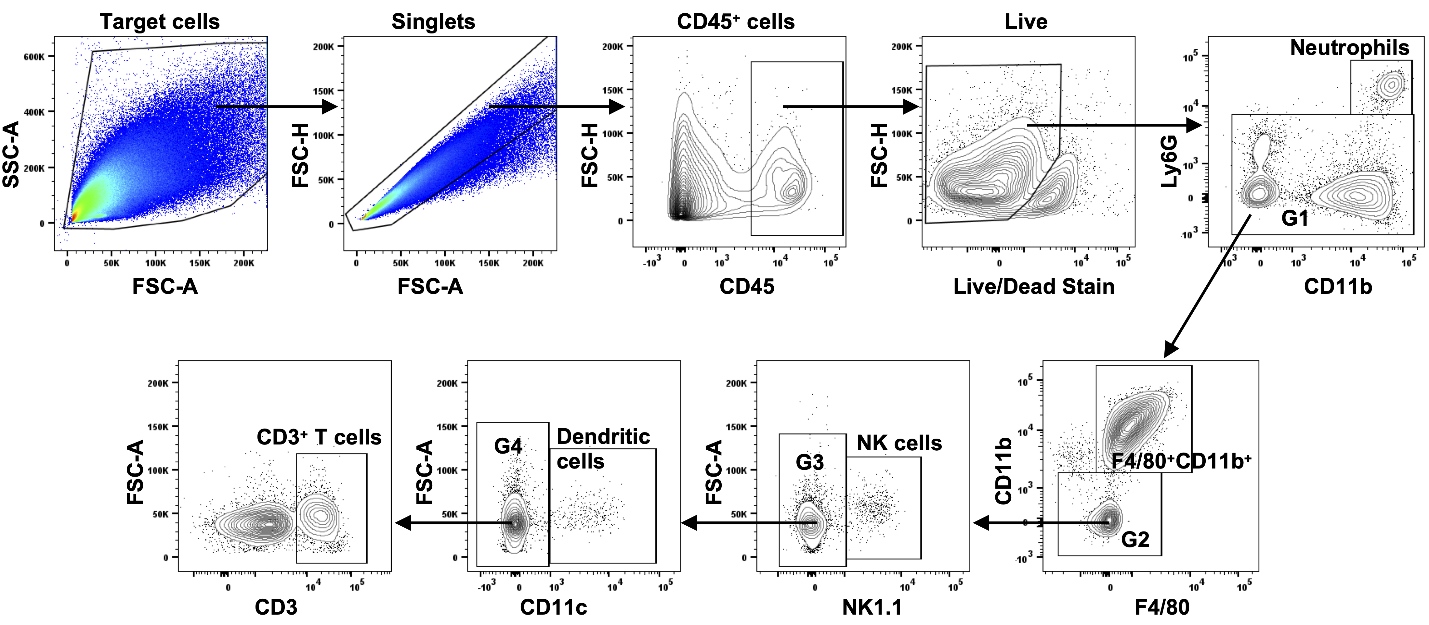
**

**Supplementary Figure 8.** Gating strategy used for the identification of Neutrophils, Macrophages, NK cells, Dendritic cells, and T cells in the heart.


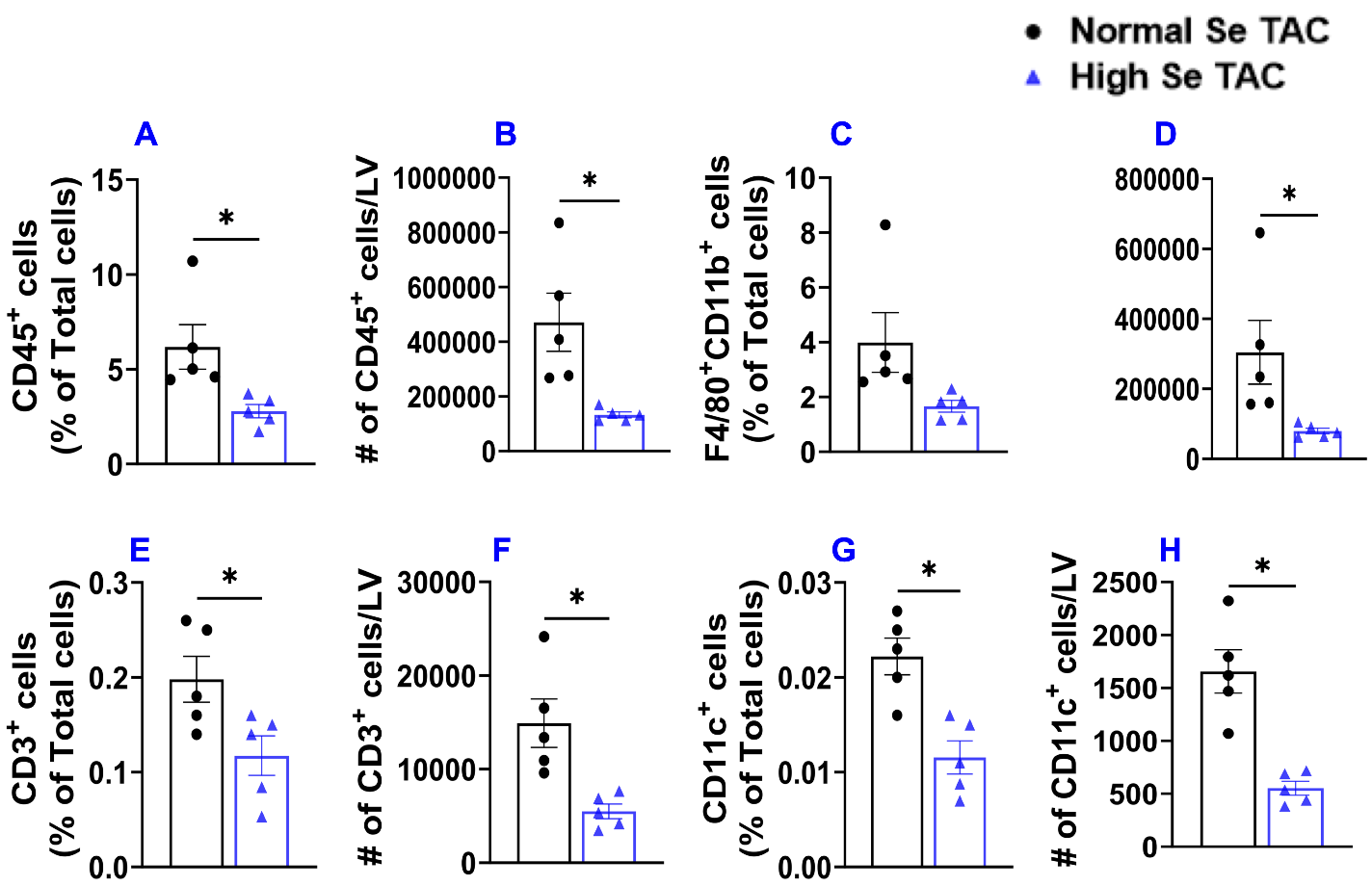


**Supplementary Figure 9.** (A, B) Quantified data of percentage of CD45^+^ leukocytes in total cells and number of CD45^+^ leukocytes per LV. (C, D) Quantified data of percentage of F4/80^+^CD11b^+^ macrophages in total cells and number of F4/80^+^CD11b^+^ macrophages per LV. (E, F) Quantified data of percentage of CD3^+^ T cells in total cells and number of CD3^+^ T cells per LV. (G, H) Quantified data of percentage of CD11c^+^ dendritic cells in total cells and number of CD11c^+^ dendritic cells per LV. *p<0.05; Se, Selenium; n=5 per group.
